# Sexuality Generates Diversity in the Aflatoxin Gene Cluster: Evidence on a Global Scale

**DOI:** 10.1371/journal.ppat.1003574

**Published:** 2013-08-29

**Authors:** Geromy G. Moore, Jacalyn L. Elliott, Rakhi Singh, Bruce W. Horn, Joe W. Dorner, Eric A. Stone, Sofia N. Chulze, German G. Barros, Manjunath K. Naik, Graeme C. Wright, Kerstin Hell, Ignazio Carbone

**Affiliations:** 1 Southern Regional Research Center, Agricultural Research Service, United States Department of Agriculture, New Orleans, Louisiana, United States of America; 2 Center for Integrated Fungal Research, Department of Plant Pathology, North Carolina State University, Raleigh, North Carolina, United States of America; 3 National Peanut Research Laboratory, Agricultural Research Service, United States Department of Agriculture, Dawson, Georgia, United States of America; 4 Bioinformatics Research Center, North Carolina State University, Raleigh, North Carolina, United States of America; 5 Department of Genetics, North Carolina State University, Raleigh, North Carolina, United States of America; 6 Departamento de Microbiología e Inmunología, Universidad Nacional de Río Cuarto, Córdoba, Argentina; 7 Department of Plant Pathology, College of Agriculture, Karnataka, India; 8 Department of Primary Industries, Kingaroy, Queensland, Australia; 9 International Institute of Tropical Agriculture, Cotonou, Republic of Benin; ETH Zurich, Switzerland

## Abstract

Aflatoxins are produced by *Aspergillus flavus* and *A. parasiticus* in oil-rich seed and grain crops and are a serious problem in agriculture, with aflatoxin B_1_ being the most carcinogenic natural compound known. Sexual reproduction in these species occurs between individuals belonging to different vegetative compatibility groups (VCGs). We examined natural genetic variation in 758 isolates of *A. flavus*, *A. parasiticus* and *A. minisclerotigenes* sampled from single peanut fields in the United States (Georgia), Africa (Benin), Argentina (Córdoba), Australia (Queensland) and India (Karnataka). Analysis of DNA sequence variation across multiple intergenic regions in the aflatoxin gene clusters of *A. flavus*, *A. parasiticus* and *A. minisclerotigenes* revealed significant linkage disequilibrium (LD) organized into distinct blocks that are conserved across different localities, suggesting that genetic recombination is nonrandom and a global occurrence. To assess the contributions of asexual and sexual reproduction to fixation and maintenance of toxin chemotype diversity in populations from each locality/species, we tested the null hypothesis of an equal number of *MAT1-1* and *MAT1-2* mating-type individuals, which is indicative of a sexually recombining population. All samples were clone-corrected using multi-locus sequence typing which associates closely with VCG. For both *A. flavus* and *A. parasiticus*, when the proportions of *MAT1-1* and *MAT1-2* were significantly different, there was more extensive LD in the aflatoxin cluster and populations were fixed for specific toxin chemotype classes, either the non-aflatoxigenic class in *A. flavus* or the B_1_-dominant and G_1_-dominant classes in *A. parasiticus*. A mating type ratio close to 1∶1 in *A. flavus*, *A. parasiticus* and *A. minisclerotigenes* was associated with higher recombination rates in the aflatoxin cluster and less pronounced chemotype differences in populations. This work shows that the reproductive nature of the population (more sexual versus more asexual) is predictive of aflatoxin chemotype diversity in these agriculturally important fungi.

## Author Summary

Fungal pathogen populations have mixed proportions of vegetative propagation and sexual reproduction ranging from predominantly clonal to varying levels of sexuality. Aflatoxins are the most potent naturally occurring carcinogens known and aflatoxin-producing *Aspergillus flavus* and *A. parasiticus* show extensive genetic and mycotoxin diversity. Population genetic studies and experimental matings in the laboratory have revealed the underlying genetic mechanisms and adaptive processes that create and maintain aflatoxin diversity. These studies provided unequivocal evidence of meiosis, crossing over, and aflatoxin heritability, but whether these processes directly influence genetic diversity in nature with respect to aflatoxin formation is not clear. Here, our work with *A. flavus*, *A. parasiticus* and *A. minisclerotigenes* from fields in different continents shows that populations with higher mean recombination rates exhibit less variability in aflatoxin profiles due to genetic intermixing, whereas populations with lower recombination rates have greater variability in aflatoxin profiles due to increased fixation of specific toxin chemotypes. Therefore, sexuality generates novel toxin chemotypes but tends to equalize toxin differences in populations. Our study highlights how an understanding of variation in mating-type frequency, fertility and recombination in these fungi is crucial for the selection of nontoxigenic biocontrol strains for long-term reduction of aflatoxins in target regions.

## Introduction

*Aspergillus flavus* and *A. parasiticus* are important fungal colonizers of food crops as well as pathogens of animals and produce the carcinogenic aflatoxins of which aflatoxin B_1_ is the most carcinogenic natural compound known [1], [2]. The two species occur in soil and drought stress in plant hosts enhances their pathogenic success [2], [3]. *A. flavus* has two recognized morphotypes that are differentiated based on sclerotial size. The L- (large) strain of *A. flavus* forms sclerotia greater than 400 µm in diameter and the S- (small) strain produces sclerotia less than 400 µm [4]. Both strains may produce B_1_+B_2_ aflatoxins (AFs) and the toxic indol-tetramic acid, cyclopiazonic acid (CPA) [5]. Aflatoxins and CPA often co-contaminate agricultural products [6]. Another species, *A. minisclerotigenes*, has the S-strain morphotype and produces both B and G aflatoxins in addition to CPA [7]. The majority of *A. parasiticus* strains also produce B and G aflatoxins but not CPA [5]; non-aflatoxigenic strains have been reported and typically accumulate *O*-methylsterigmatocystin (OMST) and dihydro-*O*-methylsterigmatocystin (DHOMST), the immediate precursors to B aflatoxins [8], [9], [10]. The loss of G aflatoxin production in *A. flavus* has been attributed to defects in, or complete absence of, the *cypA* gene that encodes cytochrome P-450 [11]. Moreover, a single point mutation can make the difference between AF+ and AF− strains [12] and partial or complete deletion of genes in AF and CPA clusters are known to exist in *A. flavus* such that strains may be AF+/CPA+, AF−/CPA−, AF+/CPA−, and AF−/CPA+ [13], [14].

Sexual reproduction in *A. flavus* L and *A. parasiticus* is heterothallic and occurs between strains of opposite mating type, either *MAT1-1* or *MAT1-2* [15], [16], [17], [18], [19]. Much of the observed heterogeneity in AF chemotype diversity in *A. flavus* and *A. parasiticus* can be attributed to intra-specific genetic exchange and recombination [18]. Genetic exchange is possible through independent assortment and crossing over during sexual reproduction or through parasexuality in heterokaryons, which are formed by the fusion of vegetatively compatible strains [20], [21]. Vegetative incompatibility among strains gives rise to vegetative compatibility groups (VCGs) that limit genetic exchange through the parasexual cycle and may eventually lead to isolation and homogeneity in toxin phenotype [22]. Aflatoxin production and morphology (sclerotium size and number; conidial color) are highly consistent within a given VCG [23]. In contrast, sexual reproduction in *A. flavus* and *A. parasiticus* occurs between individuals that belong to different VCGs and often differ in their toxigenicity [14], [16]. Experimental populations, derived from crossing sexually compatible strains in the laboratory, show high heritability of aflatoxin production in progeny strains as well as patterns of recombination in the aflatoxin cluster that mirror linkage disequilibrium (LD) in field populations [18].

In population genetic studies of a single field population in the United States, we showed that DNA sequence variation is partitioned into several distinct LD blocks across 21 intergenic regions in the aflatoxin gene clusters of *A. flavus* and *A. parasiticus* [14], [24]. Moreover, genealogical analysis of non-recombining cluster regions in *A. flavus* and *A. parasiticus* revealed trans-species polymorphisms and balancing selection acting on the non-aflatoxigenic trait in *A. flavus* [14] and on G_1_ dominant chemotypes in *A. parasiticus* [24]. In these studies, our ability to detect and estimate more frequent (or recent) recombination events in the aflatoxin cluster relied on the frequency of two or more distinct chemotype allelic classes in a population. In *A. flavus* L, DNA sequence polymorphisms in the aflatoxin gene cluster were shown to delimit two distinct evolutionary lineages named IB and IC [14], [25]. Lineage IB includes strains with partial or complete deletions of the aflatoxin cluster or full-cluster strains with many fixed polymorphisms when compared to lineage IC, which includes aflatoxigenic isolates and those that are non-aflatoxigenic due to loss-of-function mutations [14]. Lineages IB and IC are phylogenetically distinct based on DNA sequence variation across the entire aflatoxin cluster [14] and genome-wide using oligonucleotide-based array comparative genome hybridization [26]. In *A. parasiticus*, sequence variation was found to be associated with G_1_-dominant strains, which share a distinct evolutionary lineage with *A. flavus* L [24]. Recombination between divergent alleles with many fixed polymorphisms yields distinct LD blocks, whereas reduced recombination activity may be the result of a selective sweep for an advantageous chemotype or a population bottleneck that greatly reduces genetic variation [24].

The correlation between toxin chemotype profile and VCG suggests that asexual reproduction fixes diverse toxin chemotypes in populations whereas sexuality creates new VCGs with different toxin profiles. Although we expect the frequency of mating types to be close to a 1∶1 ratio in heterothallic fungi, a significant skew in the ratio does not imply a decrease in the size of the population undergoing sexual reproduction; this effective population size is also a function of the number of hermaphrodites and female sterile strains [27]. Here, we explore the contributions of asexual and sexual reproduction to mycotoxin diversity in global populations of *A. flavus*, *A. parasiticus* and *A. minisclerotigenes*. This knowledge is integral for improving biocontrol strategies worldwide and providing long-term mitigation of aflatoxin contamination in target regions.

## Materials and Methods

### Sampling, population densities and aflatoxin analyses

*Aspergillus flavus* L and S strains, *A. parasiticus* and *A. minisclerotigenes* were sampled from peanut field soils collected in different geographic regions representing five continents: United States (North America), Argentina (South America), Queensland (Australia), India (Asia), and Benin (Africa). Ecological data such as climate, peanut cultivar, and soil type were compiled for each region (Table 1). Climate data were based on compilations of monthly measurements taken 1950–2000 at weather stations closest to sampling sites (http://www.worldclim.org/). Twenty equidistant soil samples were collected along a diagonal line spanning each field. Population densities for *A. flavus* L and S, *A. parasiticus* and several other species in *Aspergillus* section *Flavi* (Table 2) were determined by dilution plating soil samples on modified dichloran-rose bengal medium and counting the number of colony-forming units (CFUs) according to Horn & Dorner [28]. Approximately four isolates each of *A. flavus* L and S and *A. parasiticus* per soil sample (when available) were single-spored by dilution plating conidia onto malt extract agar and incubating approximately 20 h at 30 C. Germlings arising from single conidia, as viewed under the light microscope at 200–400×, were then transferred to Czapek agar slants. Sample sizes for populations are shown in Table 3. Concentrations of B and G aflatoxins were determined by growing isolates on yeast extract-sucrose broth and analyzing using high performance liquid chromatography [29]. Strains were grown in 4-mL vials containing 1 mL of yeast extract sucrose broth (sucrose, 150 g; yeast extract [Difco], 20 g; soytone [Difco], 10 g; distilled water, 1 L; pH adjusted to 6.0 with HCl) for 7 d at 30°C in darkness. Vials were inoculated with approximately 1000 dry conidia and incubated under stationary conditions. Mycelial weights were not measured; replicates were incubated at the same time. *A. flavus* L and S, *A. parasiticus* and putative *A. minisclerotigenes* were then grouped into their distinct chemotype classes. The molecular evidence for distinguishing *A. flavus* S from *A. minisclerotigenes* is provided below. *A. flavus* L isolates were categorized as either aflatoxigenic (B_1_+B_2_) or non-aflatoxigenic, with non-aflatoxigenic isolates belonging to lineage IB or IC. For the S strain morphotype, chemotype classes were tentatively identified as *A. flavus* (B only) and *A. minisclerotigenes* (B+G). For *A. parasiticus* the three classes were B_1_ dominant (G_1_/B_1_≤0.5), equivalent (0.5<G_1_/B_1_<2.0) and G_1_ dominant (G_1_/B_1_≥2). We use the term “chemotype” in a broader sense to include proportionalities. Frequency distributions for distinct toxin chemotype classes were generated for *A. flavus* L and S (B_1_+B_2_), *A. parasiticus* (G_1_/B_1_), and *A. minisclerotigenes* (G_1_/B_1_) isolates from each geographic location. For *A. flavus* L, we determined the aflatoxin midpoint concentration from frequency distribution plots and the proportion of high B-producing strains (B_1_+B_2_>100 µg/mL). We graphically portrayed differences in aflatoxin concentrations for species and morphotypes from each locality using a cumulative distribution function and tested for significant differences between toxin distributions using a Kolmogorov-Smirnov test, as implemented in Matlab (MathWorks Inc., Natick, MA, USA).

**Table 1:** Climate, soil type, peanut cultivar and sampling time for each geographic region.

	Georgia/US	Córdoba/AR	Queensland/AU	Littoral/BE	Karnataka/IN
Climate	Temperate	Temperate	Semi-arid subtropical	Semi-arid subtropical	Semi-arid subtropical
Temperature (°C)^a^	30	32	32	32	32
Precipitation (mm)^b^	700	700	700	1400	1400
Soil	Tifton (sandy loam)	Franco Slimy (sandy clay)	Red Kraznozem (sandy loam)	Feralitic Clay	Rampur Series (alfisol/clay)
Peanut Cultivar	Florunner	Gran Oleico	Streeton	Chinese	Spanish
Sampling Time	Early in growing season	Early in growing season	Winter/Spring post-harvest	Immediately post-harvest	Two months post-harvest

a Approximated mean annual temperature averaged over 50 years.

b Approximated mean annual precipitation averaged over 50 years.

**Table 2:** Population soil densities for species in *Aspergillus* section *Flavi* a.

Location	*A. flavus* L	*A. flavus* S^b^	*A. parasiticus*	*A. caelatus*	*A. tamarii*	*A. alliaceus*
Argentina	31 (26.9)	2 (6.0)	37 (48.8)	8 (12.2)	-	-
India	880 (590.9)	-c	-	-	36 (47.0)	-
Benin	884 (1133.0)	7 (13.9)	-	-	61 (72.7)	-
United States	1362 (2345.0)	-	1429 (2438.0)	291 (249.0)	212 (202.0)	-
Australia	5426 (20495.6)	365 (554.2)	411 (472.4)	-	8 (25.6)	13 (33.0)

a Mean colony-forming units (CFU) and SD in parentheses per gram of dry soil weight (*n* = 20).

b *A. flavus* S and *A. minisclerotigenes* isolates not separated for CFU analysis.

c Species or morphotype not found in field soil.

**Table 3:** Population sample sizes for each geographic region.

Location	*Aspergillus flavus* L	*Aspergillus parasiticus*	*Aspergillus flavus* S	*Aspergillus minisclerotigenes*
Georgia/US	79	76	^_^ a	-
Córdoba/AR	80	80	2	2
Queensland/AU	80	80	53	27
Littoral/BE	80	-	2	42
Karnataka/IN	80	-	-	-

a Species/morphotype not found in this region.

### DNA isolation and multi-locus sequence typing (MLST)

Fungal isolates were grown on potato dextrose broth for 3–5 days at 30°C in darkness. Mycelial pellets for each isolate were harvested and freeze dried, and DNA was isolated from a single pellet as previously described [24]. PCR amplification and DNA sequencing of target loci were performed using oligonucleotide primers and thermal cycling conditions, also described previously [24]. Mating types *MAT1-1* and *MAT1-2* were determined for all isolates using multiplex-PCR [19]. All isolates were clone-corrected using DNA sequence variation at two intergenic cluster regions, *aflM/aflN* and *aflW/aflX*, and at two non-cluster loci, acetamidase (*amdS*) and tryptophan synthase (*trpC*). This MLST uniquely types approximately 84% and 59% of the VCG diversity in *A. flavus* and *A. parasiticus*, respectively [16], [18], [30]. When clone-correcting multilocus haplotypes that contained both mating types, the haplotype was counted twice as a *MAT1-1* and a *MAT1-2*. Phylogenetic reconstructions of DNA sequence variation in *aflM/aflN*, *aflW/aflX*, *MAT1-1*, *MAT1-2*, *amdS* and *trpC* were previously [31] shown to differentiate, into distinct clades, the sympatric *A. flavus* S strains that produce B aflatoxins from the S-strain morphotype isolates that produce both B and G aflatoxins in Argentina, Australia and Benin (Table 3); moreover, the S_BG_ isolates in the present study are broadly monophyletic with ex type *A. minisclerotigenes* CBS 117635 [7] and distinct from the small sclerotial *A. nomius*, *A. parvisclerotigenus* or an unnamed taxon based on variation in beta-tubulin and calmodulin (data not shown).

### Differences in mating-type frequency

Clone correction was performed to eliminate accidentally sampling the same individual multiple times or detecting epidemiological effects that do not contribute to long-term population processes. To do this, the null hypothesis of no significant difference in the frequency of *MAT1-1* and *MAT1-2* isolates for each species and geographic region was tested using a two-tailed binomial test. The test was performed on two genetic scales: the uncorrected sample and the clone-corrected sample as determined by MLST. A significant difference in mating-type frequency in the uncorrected sample but no significant difference after clone-correction or no significant difference for both uncorrected and clone-corrected samples was interpreted as primarily sexual, whereas a significant difference in mating-type frequency before and after clone-correction was interpreted as primarily asexual [27]. We used Fisher's exact test implemented in Matlab to test the relationship between 1) mating type (*MAT1-1* and *MAT1-2*) and aflatoxin chemotype class (B_1_+B_2_>0 and B_1_+B_2_ = 0 in *A. flavus* L; G_1_/B_1_≤0.5, 0.5<G_1_/B_1_<2.0, G_1_/B_1_≥2 in *A. parasiticus*), and 2) the relationship between the relative proportion of reproduction (asexual>sexual and sexual>asexual) and aflatoxin chemotype class. For *A. parasiticus* we also performed the tests assuming two broad chemotype classes (G_1_/B_1_≈1 and G_1_/B_1_≠1).

### Linkage disequilibrium and population recombination parameters in aflatoxin clusters

**Figure 1:**
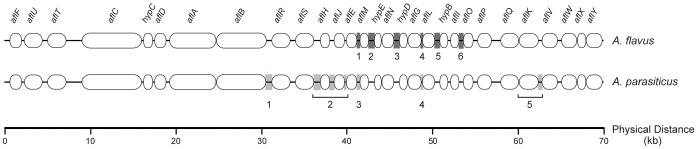
Schematic diagram of the aflatoxin gene cluster in *A. flavus* and *A. parasiticus*. The shaded blocks indicate the regions sequenced and the numbers correspond to the LD blocks outlined in Figure 3. For *A. parasiticus*, LD block 2 spans three regions and block 5 spans two regions [24].

The influence of asexual and sexual reproduction on recombination in the aflatoxin cluster and overall toxin diversity was examined by reconstructing patterns of LD in the aflatoxin cluster for a subset of isolates representing distinct MLSTs in *A. flavus* L and S, *A. parasiticus* and *A. minisclerotigenes*. Previous population genetic studies showed that multilocus cluster haplotypes are identical within a VCG and that recombination in the aflatoxin cluster is detected only between VCGs [14], [18], [24]. The subset for LD analysis was therefore selected to maximize VCG (MLST) and toxin diversity. Moreover, recombination is nonrandom and species-specific such that LD blocks and recombination hotspots are conserved among geographically separated strains [31]. We therefore determined the LD block structure and rate of recombination in the aflatoxin cluster by focusing on the intergenic regions separating LD blocks identified in the United States populations of *A. flavus* and *A. parasiticus* [14], [24]. For *A. flavus* L and S and *A. minisclerotigenes*, the regions sequenced were *aflE/aflM*, *aflM/aflN (hypE)*, *aflN/aflG*, *aflG/aflL*, *aflL/aflI*, and *aflI/aflO*, which define six distinct LD blocks [14]. For *A. parasiticus*, we sequenced *aflB/aflR*, *aflS/aflH*, *aflH/aflJ*, *aflJ/aflE*, *aflE/aflM*, *aflG/aflL*, and *aflK/aflV*, which define five LD blocks [24]. Figure 1 shows a schematic representation of the aflatoxin gene cluster and the regions that were sequenced for LD analysis. LD was examined by 1) combining all sequenced loci for each locality, species and morphotype using SNAP Combine [32] into a single concatenated sequence alignment, 2) collapsing the alignment to infer multi-locus haplotypes using SNAP Map [32] with the options of recoding indels (insertions/deletions) as binary characters and excluding infinite sites violations, and 3) generating an LD plot for all variable positions using the Clade and Matrix [33] programs implemented in SNAP Workbench [34]. LD was quantified using the coefficient of determination (*r* ^2^) between the allelic states at pairs of sites and a two-sided Fisher's Exact test, as implemented in Tassel version 1.1.0 [35]. LD blocks were based on the number of contiguous pairs of sites that were both strongly correlated (0.8<*r* ^2^<1) and significantly linked (*P*<0.01). Because highly divergent haplotypes sampled once or at a low frequency could be potential targets of balancing selection in aflatoxin gene clusters [14], [24], they were not excluded in the LD analyses and the strength of LD was assessed using both *r* ^2^ and 2×2 contingency tests. All sequences have been deposited in GenBank under Accession numbers HM353147–HM355445 and HM745560–HM745901.

For each population, we estimated the minimum number of recombination events (*R*_h_) using the RecMin program [36] and the population recombination rate per base pair using Hey and Wakeley's γ estimator, implemented in SITES version 1.1 [37]. Because cluster sequences may comprise a heterogeneous mix of highly divergent alleles [14], [24], we used the composite likelihood method and the programs convert, lkgen, interval and stat in the LDhat Version 2.2 package [38] to calculate population mean recombination rates in the aflatoxin clusters of *A. flavus* L and S, *A. parasiticus* and *A. minisclerotigenes*. The convert program was used for calculating summary statistics that included the number of segregating sites (*s*) and the average pairwise difference between sequences (π); Watterson's *θ* [39]. Tajima's *D* [40] and Fu and Li's D* [41] were used as tests of neutrality and population size constancy. The Bayesian reversible-jump Markov chain Monte Carlo (rjMCMC) scheme implemented in interval was used to estimate population mean recombination rates under a crossing-over model [18]. Before using interval, a lookup table file was created using the lkgen program for each population sample from a pre-computed table (http://ldhat.sourceforge.net/instructions.shtml) and Watterson's estimate of theta per site. The interval parameters were 1,000,000 iterations for the rjMCMC procedure; 3,500 iterations between successive samples from the chain, as recommended in the user's manual (http://ldhat.sourceforge.net/manual.pdf); and a block penalty of 0. The stat program was used to summarize the interval output for each population in terms of the upper and lower 95% confidence interval bounds on the average recombination rate across the cluster.

## Results

### Species population densities and aflatoxin concentrations

**Figure 2:**
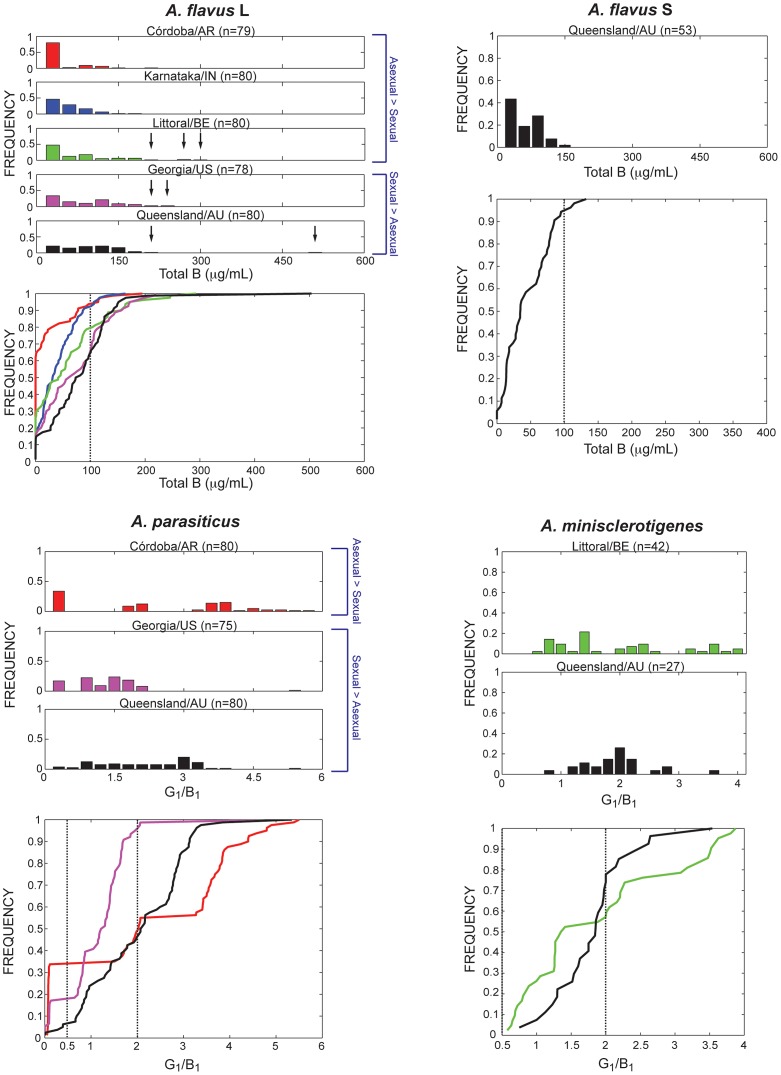
Aflatoxin frequency distributions (above) and plots of cumulative toxin distribution function (below) for *A. flavus* L and S strains, *A. parasiticus* and *A. minisclerotigenes*. Histograms use 20 bins of equal size from 0 to 600 µg/mL for *A. flavus* L and S and a ratio 0 to 6 or 0 to 4 in *A. parasiticus* and *A. minisclerotigenes*, respectively. The first bin for *A. flavus* L and S strains ranges from 0 to 30 µg/mL and comprises 60% (48/80) of non-aflatoxigenic *A. flavus* L strains in Argentina, 26% (21/80) in Benin, 18% (14/80) in India, 15% (12/79) in the United States, and 14% (11/80) in Australia; 6% (3/50) of *A. flavus* S strains in Australia were non-aflatoxigenic in the first bin. Arrows on the *A. flavus* L histograms indicate isolates having concentrations of 200 µg/mL or greater. Populations are labeled as predominantly clonal (asexual>sexual) or sexual (sexual>asexual) depending on whether there was a significant skew in the clone-corrected mating-type ratio for *A. flavus* (Table 4) and *A. parasiticus* (Table 5).

We examined a total of 758 isolates that included *A. flavus* L and S, *A. parasiticus* and *A. minisclerotigenes* sampled from five continents. *Aspergillus flavus* L was found in all regions, but *A. flavus* S, *A. parasiticus* and *A. minisclerotigenes* were not present in all sampled regions (Tables 2, 3). Across all *A. flavus* L population samples, the total concentrations of B aflatoxins generally ranged from zero to approximately 200 µg/mL, with only a few outliers in Benin, the United States and Australia having concentrations greater than 200 µg/mL (Figure 2). According to the cumulative distribution function, the percentage of *A. flavus* L isolates having a high concentration of B aflatoxins (>100 µg/mL) was skewed among localities, with Australia harboring the most toxigenic isolates with 36% (29/80) followed by the United States with 35% (27/79), Benin with 21% (17/80), India with 9% (7/80), and Argentina with 6% (5/80) (Figure 2; Table 4; Tables S1, S2, S3, S4, S5). The percentage of isolates having a low concentration of B aflatoxins (<50 µg/mL) was 83, 70, 55, 48, and 33% for Argentina, India, Benin, the United States and Australia, respectively (Figure 2). Cumulative toxin distribution functions of *A. flavus* L were not significantly different between the United States and Australia samples using a Kolmogorov-Smirnov test (*P* = 0.2201) but the United States and Australia were each significantly different from Argentina and India (*P*<0.001). The cumulative toxin distribution for Argentina also was significantly different from that of India (*P*<0.001) and Benin (*P*<0.001); however, India and Benin were not significantly different from each other (*P* = 0.0708). The Benin toxin distribution was significantly different from that of Australia (*P* = 0.002) but not significantly different from the United States (*P* = 0.1061). Total aflatoxin midpoint concentrations from the United States and Australia were 60 and 80 µg/mL, respectively, whereas in Argentina, India and Benin midpoint concentrations were only 0, 30 and 40 µg/mL, respectively (Table 4). Approximately 60% (48/80) of the *A. flavus* L isolates sampled in Argentina were non-aflatoxigenic (Figure 2, Table S1), and 35 out of the 48 non-aflatoxigenic isolates (73%) belonged to lineage IB. By comparison, the India and Benin population samples contained four and two *A. flavus* L isolates, respectively, in lineage IB and only singletons from this lineage were found in the United States and Australia (Table 4).

**Table 4:** Aflatoxin and mating-type distribution in populations of *A. flavus* L strain with different proportions of asexual and sexual reproduction.

Region	AF midpoint^a^ (µg/mL)	High B AF^b^	Lineage IB^c^	Genetic Scale^d^	Mating-type Frequency^e^		*P*-value^f^
					*MAT1-1*	*MAT1-2*	
**Asexual>Sexual**							
Córdoba/AR	0	5/80	35/80	Uncorrected	85 (67)	15 (12)	<0.0001
				**Haplotype corrected**	**76 (19)**	**24 (6)**	**0.0146**
Karnataka/IN	30	7/80	4/80	Uncorrected	63 (50)	37 (30)	0.0330
				**Haplotype corrected**	**69 (25)**	**31 (11)**	**0.0288**
Littoral/BE	40	17/80	2/80	Uncorrected	61 (49)	39 (31)	0.0567
				**Haplotype corrected**	**64 (42)**	**36 (23)**	**0.0248**
**Sexual>Asexual**							
Georgia/US	60	27/79	1/79	Uncorrected	19 (15)	81 (63)	<0.0001
				**Haplotype corrected**	**38 (15)**	**62 (24)**	**0.1996**
Queensland/AU	80	29/80	1/80	Uncorrected	37 (29)	63 (50)	0.0238
				**Haplotype corrected**	**37 (13)**	**63 (22)**	**0.1755**

a AF concentration (B_1_+B_2_) midpoint (µg/mL) for frequency distribution plots in Figure 2.

b Number of isolates having AF (B_1_+B_2_)>100 µg/mL out of the total isolates in sample.

c Number of AF− isolates out of the total number of full-cluster isolates that group with Geiser's group IB (25) based on phylogenetic inference for *aflW/aflX* region.

d Samples either uncorrected or haplotype corrected (bold type) based on four genomic loci: *aflM/aflN*, *aflW/aflX*, *amdS*, *trpC*.

e Numbers in parentheses refer to number of isolates (uncorrected) or haplotypes (corrected) examined for each genetic scale.

f Probability of a binomial test (two-tailed). Test was performed on the raw data.

In *A. parasiticus* populations, the frequencies of the three chemotype classes B_1_ dominant, G_1_/B_1_ equivalent and G_1_ dominant differed significantly among localities (Figure 2). The Argentina sample (n = 80) had more G_1_- and B_1_-dominant isolates (41 and 27, respectively) than G_1_/B_1_ equivalent isolates (12) (Table 5) and the distribution of G_1_/B_1_ was approximately partitioned into three chemotype classes (Figure 2). By contrast, the United States sample showed significantly more G_1_/B_1_ equivalent isolates (n = 59) than G_1_- and B_1_-dominant isolates (4 and 9, respectively) (Table 5). In Australia, the G_1_-dominant and G_1_/B_1_ equivalent chemotype classes (43 and 32, respectively) predominated over B_1_-dominant isolates (n = 4) (Table 5). The G_1_/B_1_ ratio for Australia and the United States showed a unimodal distribution (Figure 2), and cumulative toxin distribution functions for Argentina, the United States and Australia (Figure 2, Tables S6, S7, S8) were significantly different from each other using a Kolmogorov-Smirnov test (*P*<0.0001).

**Table 5:** Aflatoxin and mating-type distribution of *A. parasiticus* with different proportions of asexual and sexual reproduction.

Region	B_1_>>G_1_ a	G_1_ = B_1_ b	G_1_>>B_1_ c	Genetic Scale^d^	Mating-type Frequency^e^		*P*-value^f^
					*MAT1-1*	*MAT1-2*	
**Asexual>Sexual**							
Córdoba/AR	27	12	41	Uncorrected	98 (78)	2 (2)	<0.0001
				**Haplotype corrected**	**88 (15)**	**12 (2)**	**0.0023**
**Sexual>Asexual**							
Georgia/US^g^	9	59	4	Uncorrected	81 (61)	19 (14)	<0.0001
				**Haplotype corrected**	**72 (13)**	**28 (5)**	**0.0963**
Queensland/AU^h^	4	32	43	Uncorrected	38 (29)	62 (48)	0.0395
				**Haplotype corrected**	**41 (12)**	**59 (17)**	**0.4582**

a Number of isolates that are B_1_ dominant (G_1_/B_1_≤0.5).

b Number of isolates that have equivalent amounts of G_1_ and B_1_ (0.5<G_1_/B_1_<2.0).

c Number of isolates that are G_1_ dominant (G_1_/B_1_≥2.0).

d Samples either uncorrected or haplotype corrected (bold type) based on four genomic loci: *aflM/aflN*, *aflW/aflX*, *amdS*, *trpC*.

e Numbers in parentheses refer to number of isolates (uncorrected) or haplotypes (corrected) examined for each genetic scale.

f Probability of a binomial test (two-tailed). Test was performed on the raw data.

g Four isolates produce only OMST at 80–250 µg/mL.

h One isolate produces only OMST at 119 µg/mL.

Populations sampled from Australia (*A. flavus* S and *A. minisclerotigenes*) and Benin (*A. minisclerotigenes*) were partitioned into their respective chemotype classes B and B+G (Table 6). The cumulative toxin frequency distribution for *A. flavus* S from Australia showed that approximately 6% (3/50) of the isolates had a high concentration of B aflatoxins (>100 µg/mL), which was significantly different (*P*<0.0001) from the 36% (29/80) of L strains from Australia with high B aflatoxins (Figure 2). The *A. minisclerotigenes* toxin distributions (Figure 2; Table 6) were not significantly different between Australia and Benin (*P* = 0.076). The toxin profiles for all *A. flavus* S and *A. minisclerotigenes* strains are found in Tables S9, S10, S11.

**Table 6:** Aflatoxin and mating-type distribution *Aspergillus flavus* S strain and *A. minisclerotigenes*.

Region	Aflatoxin Profile				Genetic Scale^e^	Mating-type Frequency^f^		*P*-value^g^
	*A. flavus* S	*A. minisclerotigenes*				*MAT1-1*	*MAT1-2*	
	B^a^	B_1_>G_1_ b	G_1_ = B_1_ c	G_1_>B_1_ d				
Queensland/AU^h^	50	–	–	–	Uncorrected	18 (6)	82 (28)	0.0002
					**Haplotype corrected**	**38 (10)**	**62 (16)**	**0.3269**
	–	0	19	8	Uncorrected	88 (7)	12 (1)	0.0703
					**Haplotype corrected**	**83 (5)**	**17 (1)**	**0.2188**
Littoral/BE	–	0	24	18	Uncorrected	43 (9)	57 (12)	0.6636
					**Haplotype corrected**	**40 (6)**	**60 (9)**	**0.6072**

a Number of isolates that produce B (B_1_+B_2_) AFs only.

b Number of isolates that are B_1_ dominant (G_1_/B_1_≤0.5).

c Number of isolates that have equivalent amounts of G_1_ and B_1_ (0.5<G_1_/B_1_<2.0).

d Number of isolates that are G_1_ dominant (G_1_/B_1_≥2.0).

e Samples either uncorrected or haplotype corrected (bold type) based on four genomic loci: *aflM/aflN*, *aflW/aflX*, *amdS*, *trpC*.

f Numbers in parentheses refer to number of isolates (uncorrected) or haplotypes (corrected) examined for each genetic scale.

g Probability of a binomial test (two-tailed). Test was performed on the raw data.

h Three isolates are non-aflatoxigenic, producing neither B nor G aflatoxins.

### Differences in mating-type frequency

There was a significant disparity in the number of *MAT1-1* and *MAT1-2* isolates of *A. flavus* L in the Argentina, India and Benin populations, with *MAT1-1* being the dominant mating type for both the uncorrected and MLST-corrected samples (Table 4). In the United States and Australia, *MAT1-2* was more abundant than *MAT1-1* in the uncorrected samples, but this difference was not significant after clone correction (Table 4). Mating-type ratios were also skewed in favor of *MAT1-1* in *A. parasiticus* populations sampled from Argentina and the United States, whereas *MAT1-2* predominated in Australia (Table 5). In Argentina, 98% (78/80) of the isolates were *MAT1-1*; clone correction of 63 *MAT1-1* strains yielded 15 multilocus haplotypes with 10 haplotypes represented only once. The largest *MAT1-1* multilocus haplotype comprised 29 strains. In contrast, clone correction of the United States and Australia samples of *A. parasiticus* showed that differences in *MAT1-1* and *MAT1-2* were not significant (*P* = 0.0963 and 0.4582, respectively). Similarly, mating-type ratios showed no significant deviation from 1.0 in clone-corrected samples of *A. flavus* S and *A. minisclerotigenes* from Australia and Benin (Table 6).

In *A. flavus* L, there was a significant association between mating types (*MAT1-1* and *MAT1-2*) and the two aflatoxin chemotype classes (B_1_+B_2_ = 0 and B_1_+B_2_>0) in Argentina (*P*<0.001; Table S1) using a Fisher's exact test; however, there was no significant association between mating type and chemotype classes in India (*P* = 1.0; Table S2), Benin (*P* = 0.4327; Table S3), United States (*P* = 0.4725; Table S4), and Australia (*P* = 0.4246; Table S5). In *A. parasiticus*, there were not enough data to observe an association between mating type and the three chemotype classes (G_1_/B_1_≤0.5, 0.5<G_1_/B_1_<2.0, G_1_/B_1_≥2) in Argentina (Table S6) and there was no relationship between mating type and chemotype in the United States (Table S7) and Australia (Table S8). By comparison, in *A. flavus* L populations (Tables S1, S2, S3, S4, S5), the two aflatoxin chemotype classes (B_1_+B_2_ = 0 and B_1_+B_2_>0) were significantly (*P*<0.0001) associated with the relative proportion of reproduction (asexual>sexual and sexual>asexual). Similarly, in *A. parasiticus* populations (Table S6, S7, S8), the three aflatoxin chemotype classes (G_1_/B_1_≤0.5, 0.5<G_1_/B_1_<2.0, G_1_/B_1_≥2) were significantly (*P*<0.0001) associated with the relative proportion of reproduction (asexual>sexual and sexual>asexual; Table 5); there was also a significant (*P*<0.0001) association of the latter with two broad chemotype classes (G_1_/B_1_≈1 and G_1_/B_1_≠1).

### Linkage disequilibrium and population recombination parameters in aflatoxin gene clusters

**Figure 3:**
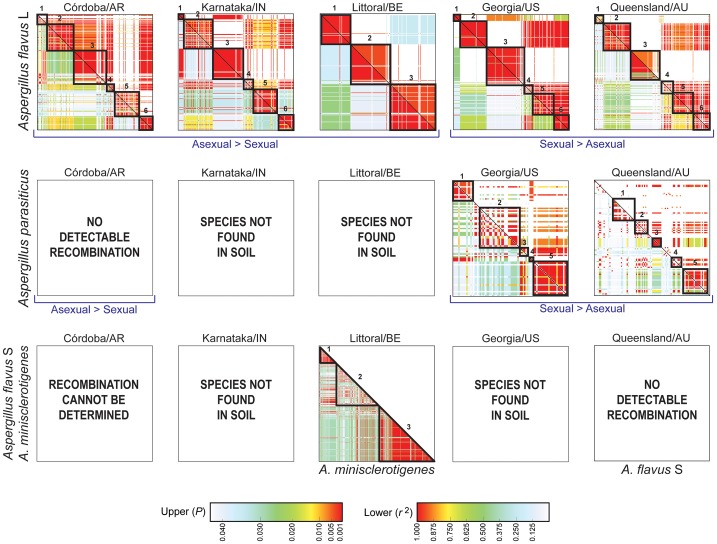
LD plots in which the upper triangular matrix represents the *P* values calculated using Fisher's Exact test; the lower triangular matrix represents *r* ^2^, the coefficient of determination between allelic states at pairs of sites. Colored shading in the LD plot indicates statistical significance in linkage (*P*<0.01) and strength of associations (0.8<*r* ^2^<1) among sites. In the reference population from the United States, there are six and five distinct LD blocks (outlined squares) in *A. flavus* L and *A. parasiticus*, respectively, based on *P* and *r^2^*. *Aspergillus parasiticus* was not found in India and Benin; *A. flavus* S and *A. minisclerotigenes* were not found in India and the United States and infrequently sampled in Argentina such that recombination could not be determined.

Sympatric populations of *A. flavus* L and S, *A. parasiticus* and *A. minisclerotigenes* were sampled only from Australia and Argentina (Table 3). In *A. flavus* L, patterns of LD in the aflatoxin gene cluster were conserved across all populations but there were differences in the size of LD blocks and recombination parameters (Figure 3; Table 7). While the six distinct blocks observed in the United States can also be discerned in Australia, Argentina and India, blocks 4, 5 and 6 were merged into a single LD block in Benin (Figure 3). The Benin *A. flavus* L population with three distinct blocks showed the most extensive LD in the cluster (Figure 3), also evidenced by the lowest population mean recombination rate (2*N_e_r; ρ* = 0.0006), the lowest recombination rate per base pair (γ = 0.0002) and smallest minimum number of inferred recombination events (*R*_h_ = 1) (Table 7). The minimum number of recombination events and rates were similar in the other two predominantly clonal *A. flavus* L populations in India (*ρ* = 0.0069, γ = 0.0016, *R*_h_ = 5) and Argentina (*ρ* = 0.0026, γ = 0.0024, *R*_h_ = 7). The predominantly sexual *A. flavus* L populations in the United States and Australia harbored an almost identical LD block structure (Figure 3), and isolates from both locations were similar in their aflatoxin concentrations (Table 4), recombination rate estimates (γ = 0.0011 and 0.0010, respectively) and minimum number of recombination events (*R*_h_ = 6 and 5, respectively) (Table 7). The positive and non-significant values of Tajima's *D* and Fu & Li D* tests indicated the presence of divergent alleles and balancing selection on aflatoxin production and non-production in *A. flavus* L aflatoxin clusters (Figure S1) [14]. Estimates of π and *θ* were very similar across all *A. flavus* L populations, which indicate no significant underlying differences in mutation rates and population genetic structure.

**Table 7:** Diversity, neutrality, and recombination in populations of *A. flavus* L and S strains, *A. parasiticus* and *A. minisclerotigenes*.

Region	*n* a	π^b^	*θ* c	Tajima's *D* d	Fu & Li D*^e^	*s* f	*R*_h_ g	γ^h^	2*N_e_r* i
Córdoba/AR									
*A. flavus* L	19 (9, 10)	0.0435	0.0302	1.849	1.014	273	7	0.0024	0.0026 (0.0024, 0.0034)
*A. parasiticus*	20	0.0013	0.0036	−2.561	−4.029	41	0	n/e^j^	n/e
Karnataka/IN									
*A. flavus* L	20 (4, 16)	0.0383	0.0272	1.688	1.513	244	5	0.0016	0.0069 (0.0036, 0.0144)
Littoral/BE									
*A. flavus* L	13 (0, 13)	0.0365	0.0283	1.322	1.569	222	1	0.0002	0.0006 (0.0004, 0.0015)
*A. minisclerotigenes*	6	0.0554	0.0520	0.427	0.327	397	4	0.0028	0.0108 (0.0051, 0.0235)
Georgia/US									
*A. flavus* L	43 (1, 42)	0.0312	0.0227	1.379	1.493	253	5	0.0011	0.1114 (0.0755, 0.1808)
*A. parasiticus*	24	0.0074	0.0075	−0.056	0.329	83	4	0.0016	0.0049 (0.0039, 0.0063)
Queensland/AU									
*A. flavus* L	19 (1, 18)	0.0364	0.0312	0.707	0.380	277	6	0.0010	0.0286 (0.0214, 0.0354)
*A. flavus* S	7	0.0120	0.0157	−1.394	−1.572	121	0	0.0000	0.0005 (0.0003, 0.0010)
*A. parasiticus*	19	0.0106	0.0109	−0.107	−0.464	126	8	0.0099	0.0285 (0.0049, 0.0912)

a Numbers of *A. flavus* L strain; lineage IB and IC isolates included in LD analysis for Figure 3 are shown in parentheses. Sample sequences based on six genomic loci (*aflE/aflM, aflM/aflN, aflN/aflG, aflG/aflL, aflL/aflI, aflI/aflO*) in *A. flavus* L, S and *A. minisclerotigenes*, and seven loci (*aflB/aflR, aflS/aflH, aflH/aflJ, aflJ/aflE, aflE/aflM, aflG/aflL, aflK/aflV*) in *A. parasiticus*.

b Average pairwise differences between nucleotides across multiple cluster loci per site.

c Watterson's estimator of the population-scaled mutation rate per site.

d Tajima's *D* statistic is a measure of departure from neutrality.

e Fu and Li D* statistic is a measure of the departure of the frequency spectrum from neutral expectations.

f Number of segregating sites across multiple concatenated cluster loci.

g Minimum number of recombination events across multiple concatenated cluster loci using RecMin.

h Population recombination rate estimate per base pair.

i Population mean recombination rate per site. First value is mean *ρ* and the numbers in parentheses are the lower and upper 95% confidence intervals, respectively.

j Population sample data are fully compatible and recombination cannot be determined.

In *A. parasiticus*, the five LD blocks identified in the United States were not as distinct in Australia and only blocks 4 and 5 were detected; the largest LD block in the United States (block 2) was further split into two blocks in Australia (Figure 3). The population mean recombination rate in the aflatoxin cluster was six-fold higher in Australia than in the United States (*ρ* = 0.0285 and 0.0049, respectively) and a similar trend was observed in overall estimates of recombination rate per base pair (γ = 0.0099 and 0.0016, respectively) and minimum number of recombination events (*R*_h_ = 8 and 4, respectively) (Table 7). No recombination was detected in the Argentina *A. parasiticus* population (Figure 3, Table 7). In all cases, populations of *A. parasiticus* with higher recombination rates had more segregating sites in the cluster (Table 7). The negative values of Tajima's *D* and Fu & Li D* indicated a reduction of genetic variation across the entire cluster (Figure S2). This was most pronounced in the *A. parasiticus* population from Argentina (π = 0.0013, *θ* = 0.0036), which is highly clonal based on mating-type distributions (Table 5). Population parameter estimates and neutrality tests for *A. flavus* S in Australia (π = 0.0120, *θ* = 0.0157, *s* = 121) were very similar to those for sympatric *A. parasiticus* (π = 0.0106, *θ* = 0.0109, *s* = 126) (Table 7). By contrast, *A. minisclerotigenes* cluster population parameters in Benin (π = 0.0554, *θ* = 0.052, *s* = 397) were approximately double those of sympatric *A. flavus* L (π = 0.0365, *θ* = 0.0283, *s* = 222), with a population mean recombination rate (*ρ* = 0.0108) in *A. minisclerotigenes* that was several orders of magnitude larger than that of sympatric *A. flavus* L (*ρ* = 0.0006) and with resolution of only a single LD block comprising *A. flavus* L blocks 4, 5 and 6 (Figures 3 and S3).

## Discussion

In heterothallic and hermaphroditic fungal species, mating type segregates as a single Mendelian locus such that a 1∶1 ratio is expected in a sexually reproducing population [27]. The results from this study indicate that the proportion of clone-corrected *MAT1-1* and *MAT1-2* in populations of *A. flavus* L and *A. parasiticus* is a useful indicator and predictor of whether populations are more clonal or sexual in reproduction. Moreover, the reproductive nature of the population (more sexual versus more asexual) is predictive of aflatoxin chemotype, in that predominantly asexual populations show a larger proportion of non-aflatoxigenic *A. flavus* L and an excess of G_1_- and B_1_-dominant *A. parasiticus* clones. There were too few data points (one per field per species) to directly test whether mating type frequency correlates with aflatoxin chemotypes; however, we were able to test the relationship between the relative proportion of sexual versus asexual reproduction and chemotype diversity. Overall, sexuality generates novel toxin chemotypes but tends to equalize toxin differences in populations. Sexual populations of *A. flavus*, *A. parasiticus* and *A. minisclerotigenes* from fields in different continents showed less variability in aflatoxin profiles due to genetic intermixing, whereas asexual populations exhibited greater variability in aflatoxin profiles due to increased fixation of specific toxin chemotypes.

In *A. flavus* L, a significant skew in the mating-type ratio was associated with higher recombination rates in the aflatoxin gene cluster and less pronounced chemotype differences. Predominantly asexual *A. flavus* L populations had lower mean recombination rates in the aflatoxin gene cluster, a larger proportion of non-aflatoxigenic clones and larger LD blocks. Although the size of LD blocks varied in asexual populations, block boundaries were conserved among different localities, suggesting a nonrandom distribution of recombination hotspots, as reported in other fungi [42]; infrequent recombination would initially give rise to larger LD blocks and as recombination rates increase there would be a gradual erosion of LD and more blocks that coincide with recombination hotspots. For example, overall estimates of population mean recombination rates in *A. flavus* L were 12-fold (0.0069/0.0006) larger in India and 4-fold (0.0026/0.0006) larger in Argentina than in Benin, which had only three LD blocks spanning the same physical distance (Figure 3; Table 7). Although *A. flavus* L is predominantly clonal in India, Argentina and Benin (Table 4), the ratio of asexual∶sexual reproduction is highest in Benin. By contrast, mean recombination rates in predominantly sexual *A. flavus* L populations (United States, Australia) were on average 23-fold (0.07/0.003) larger than in asexual populations (Argentina, India, Benin). Low recombination rates were also associated with distinct aflatoxin chemotype classes that included a relatively high frequency of non-aflatoxigenic clones (Figure 2). Approximately 60% (48/80) of the *A. flavus* L strains in Argentina were non-aflatoxigenic, followed by 26% (21/80) in Benin, 18% (14/80) in India, 15% (12/79) in the United States, and 14% (11/80) in Australia. Overall, *A. flavus* L populations with a mating type ratio closer to 1∶1 had higher population mean recombination rates, which translated into more recombination between non-aflatoxigenic and predominantly aflatoxigenic strains, thereby equalizing chemotype differences, as observed in laboratory crosses [18].

In Argentina, a broad sampling of *A. flavus* L from peanut seeds and soil revealed approximately 49% were non-aflatoxigenic with 13% harboring deletions of aflatoxin cluster genes (S. N. Chulze, personal communication), which suggests that lineage IB may be more prevalent than lineage IC. In this case, clonal proliferation as a result of directional selection on non-aflatoxigenicity may preserve lineage IB whereas sex between lineages IB and IC will increase the proportion of new genotypes that are aflatoxigenic, as demonstrated in *A. flavus* L populations derived from experimental matings [18]. Similarly, the lower recombination rate of *A. flavus* L in Benin may not necessarily be the result of lower recombination rates *per se*, but instead a paucity of sexually fertile lineage IB strains that would allow us to track recombination events when they occur. As seen in Tables 4 and 7, when the number of *A. flavus* L isolates in lineage IB increases from two in Benin to 35 in Argentina (*n* = 80), there is a four-fold increase in the rate of recombination (*ρ*) and a seven-fold increase in the minimum number of recombination events (*R*_h_) in the cluster. Despite differences in population mean recombination rates, nucleotide diversity (π) and population mutation rate parameter (*θ*) were similar in magnitude, which suggests that divergent IB and IC alleles exist in all populations, but limited recombination results in extensive LD in the aflatoxin cluster (Figs. 3, 1S). For example, even though *A. flavus* L in Argentina and India showed an LD block structure similar to that observed in the United States and Australia, contingency testing revealed stronger LD in Argentina (see upper diagonal matrix in Figure 3) than in India. This suggests mating type ratio alone is not a good predictor of LD patterns in the aflatoxin cluster. In the absence of sex, non-aflatoxigenic strains may have an advantage over aflatoxigenic strains during vegetative growth or clonal populations in more temperate latitudes may be disproportionate for lineage IB isolates and therefore favor non-aflatoxigenicity. There may also be an ecological cost to aflatoxin production in certain environments depending on the level of competition or stress, such that alleviating competition favors non-aflatoxigenicity.

In *A. parasiticus*, a significant skew in the mating-type ratio was also correlated with both qualitative and quantitative differences in aflatoxin production that included a relatively high frequency of isolates in B_1_-dominant and G_1_-dominant classes. For example, *A. parasiticus* in Argentina was predominantly clonal based on mating-type frequencies; moreover, there was no detectable recombination in the aflatoxin cluster and the G_1_/B_1_ toxin distribution showed an excess of G_1_- and B_1_-dominant isolates (Figure 2), possibly the result of disruptive selection for B_1_- and G_1_-dominant traits. The lack of recombination in the *A. parasiticus* population from Argentina may have driven the fixation of both B_1_- and G_1_-dominant chemotypes. Alternatively, there may have been a recent selective sweep of the *MAT1-1* mating type acting on B_1_ and G_1_ dominant chemotypes. In contrast, the predominantly sexual *A. parasiticus* populations in the United States and Australia showed higher recombination rates, distinct LD blocks in the cluster and a greater proportion of the equivalent chemotype class (0.5<G_1_/B_1_<2.0). The equivalent G_1_/B_1_ ratios in sexual populations suggest mating between parents that are high and low producers, resulting in progeny strains with intermediate toxicities of parental strains, as observed in experimental crosses [26]. Moreover, strains of *A. parasiticus* accumulating *O*-methylsterigmatocystin (OMST) were only found in sexual populations, suggesting that another outcome of sex in *A. parasiticus* may be to increase chemotype diversity. Because OMST accumulation results from the substitution of a single amino acid residue in *aflQ* [10], which is immediately adjacent to block 5 in *A. parasiticus* (Figure 1), it is plausible that more sexual reproduction will increase the probability of transferring this mutation to other strains via crossing over in the aflatoxin cluster. Alternatively, there may have been trans-species evolution as previously reported [24] such that *A. flavus* L and *A. parasiticus* OMST-accumulating and G_1_-dominant strains share a recent common ancestor, which may also be indicative of hybridization. In *A. flavus* L and *A. parasiticus*, fertile crosses comprise parents belonging to different VCGs [15], [17] and it is possible that inter-specific barriers to hyphal fusion may also be suppressed during inter-specific mating. This supports an earlier observation that *A. flavus* and *A. parasiticus* show a high degree of genome similarity that is comparable to other inter-fertile species [43] and points to the possibility of hybridization in nature, which has been shown to be experimentally feasible [44]. Because *A. minisclerotigenes* strains are more similar to *A. parasiticus* than *A. flavus* L in terms of B and G aflatoxin production and the existence of G_1_-dominant strains, we hypothesize that *A. minisclerotigenes* and *A. parasiticus* aflatoxin clusters are under similar evolutionary constraints; for example, both have an intact *aflF/aflU* intergenic region necessary for G aflatoxin production [45]. In this paper chemotypes are phenotypic groupings. It is possible that B+G toxin groups may be associated with genetic differences in the aflatoxin cluster that do not necessarily include the specific genes (e.g., *aflU*) directly responsible for mycotoxin profiles.

A skew in the mating-type ratio may be indicative of other processes such as genetic drift due to female sterility that can shift populations toward clonality; if the frequency of sex in populations is low, then the signature of clonality should be detectable. For the sympatric *A. parasiticus* and *A. flavus* populations in the United States, the uncorrected mating-type distributions are significantly skewed in opposite directions such that *A. parasiticus* has a higher frequency of *MAT1-1* and *A. flavus* has a higher frequency of *MAT1-2*, although these differences are not significant after clone correction. This differential skew in the uncorrected samples in the United States may be driven by species-specific differences in fertility such that a greater proportion of the fertile females are *MAT1-2* in *A. flavus* and *MAT1-1* in *A. parasiticus*, but this cannot be ascertained without further mating studies. Alternatively, a higher frequency of one mating type may be the result of increased fitness on a function other than mating. The mating-type genes *MAT1-1* and *MAT1-2* encode putative transcription factors regulating pheromone and pheromone receptor genes as well as other genes not involved directly in the mating process [27]. The dominance of *MAT1-2* in *A. flavus* L sexual populations in the United States and Australia suggest that populations can have an overriding clonal component despite undergoing sex [46]. There was also evidence of sex in clonal populations of *A. flavus* L from Argentina, India and Benin. Clonal populations of *A. flavus* L overall were predominantly *MAT1-1* even though these fungi were sampled from diverse soil ecologies and exposed to different environmental conditions (Table 1). Sampling more fields in different geographical regions will be necessary to fully understand the role of different ecological and environmental factors on aflatoxin production.

Understanding the underlying genetic processes that generate diversity in *A. flavus* and *A. parasiticus* populations has direct implications in biological control in which competitive non-aflatoxigenic strains of *A. flavus* are applied to crops to reduce aflatoxin contamination [47]. Our observation that aflatoxin chemotype diversity in a population is associated with the reproductive nature of the population (more sexual versus more asexual) can be useful in fine-tuning biocontrol to the underlying population dynamics of a specific field. We expect that more sexual populations will exhibit higher mean rates of recombination in the aflatoxin cluster and display a more unimodal distribution of toxin concentrations. For example, Argentina is a mostly clonal population for both *A. flavus* and *A. parasiticus*, and *MAT1-1* greatly outnumbers *MAT1-2* even after clone correction. An indigenous non-aflatoxigenic isolate that is *MAT1-1* might be recommended as a biocontrol agent in such a field, since the potential to recombine with indigenous *MAT1-2* toxin producers is relatively low; however, the degree of fertility of the introduced strain may also be an important consideration and in this case, the number of distinct VCGs in the field and their fertility as deduced from laboratory crosses, may be more informative for biocontrol. In contrast, the frequency of *MAT1-1* and *MAT1-2* isolates for *A. flavus* and *A. parasiticus* in the Australia field was approximately 1∶1 even after clone correction. Under such circumstances, the potential of a biocontrol strain for recombining with a toxin producer is greater and approaches that focus on other biological traits, such as female sterility, may be more effective.

## Supporting Information

Figure S1 Distribution of SNPs and indels among haplotypes for each *A. flavus* L-strain population. Vertical lines correspond to putative boundaries for distinct LD blocks shown in Figure 3.
(TIF)

Figure S2 Distribution of SNPs and indels among haplotypes for each *A. parasiticus* population. Vertical lines correspond to putative boundaries for distinct LD blocks shown in Figure 3. There was no evidence of recombination in the Argentina population and the entire region examined falls into a single LD block.
(TIF)

Figure S3 Distribution of SNPs and indels among haplotypes for each *A. flavus* S-strain and *A. minisclerotigene* population in Australia and Benin, respectively. Vertical lines correspond to putative boundaries for distinct LD blocks shown in Figure 3. There was no evidence of recombination in *A. flavus* S sampled in Australia.
(TIF)

Table S1 *Aspergillus flavus* L isolates from Córdoba, Argentina.
(DOC)

Table S2 *Aspergillus flavus* L isolates from Karnataka, India.
(DOC)

Table S3 *Aspergillus flavus* L isolates from Littoral, Benin.
(DOC)

Table S4 *Aspergillus flavus* L isolates from Georgia, United States.
(DOC)

Table S5 *Aspergillus flavus* L isolates from Queensland, Australia.
(DOC)

Table S6 *Aspergillus parasiticus* isolates from Córdoba, Argentina.
(DOC)

Table S7 *Aspergillus parasiticus* isolates from Georgia, United States.
(DOC)

Table S8 *Aspergillus parasiticus* isolates from Queensland, Australia.
(DOC)

Table S9 *Aspergillus flavus* S and *A. minisclerotigenes* isolates from Queensland, Australia.
(DOC)

Table S10 *Aspergillus flavus* S and *A. minisclerotigenes* isolates from Littoral, Benin.
(DOC)

Table S11 *Aspergillus flavus* S and *A. minisclerotigenes* isolates from Córdoba, Argentina.
(DOC)
