# Supplementary figures and images for: Sexuality Generates Diversity in the Aflatoxin Gene Cluster: Evidence on a Global Scale
Source: PLoS Pathog. 2013 Aug 29;9(8):e1003574. doi: 10.1371/journal.ppat.1003574 (PMC3757046; doi:10.1371/journal.ppat.1003574)

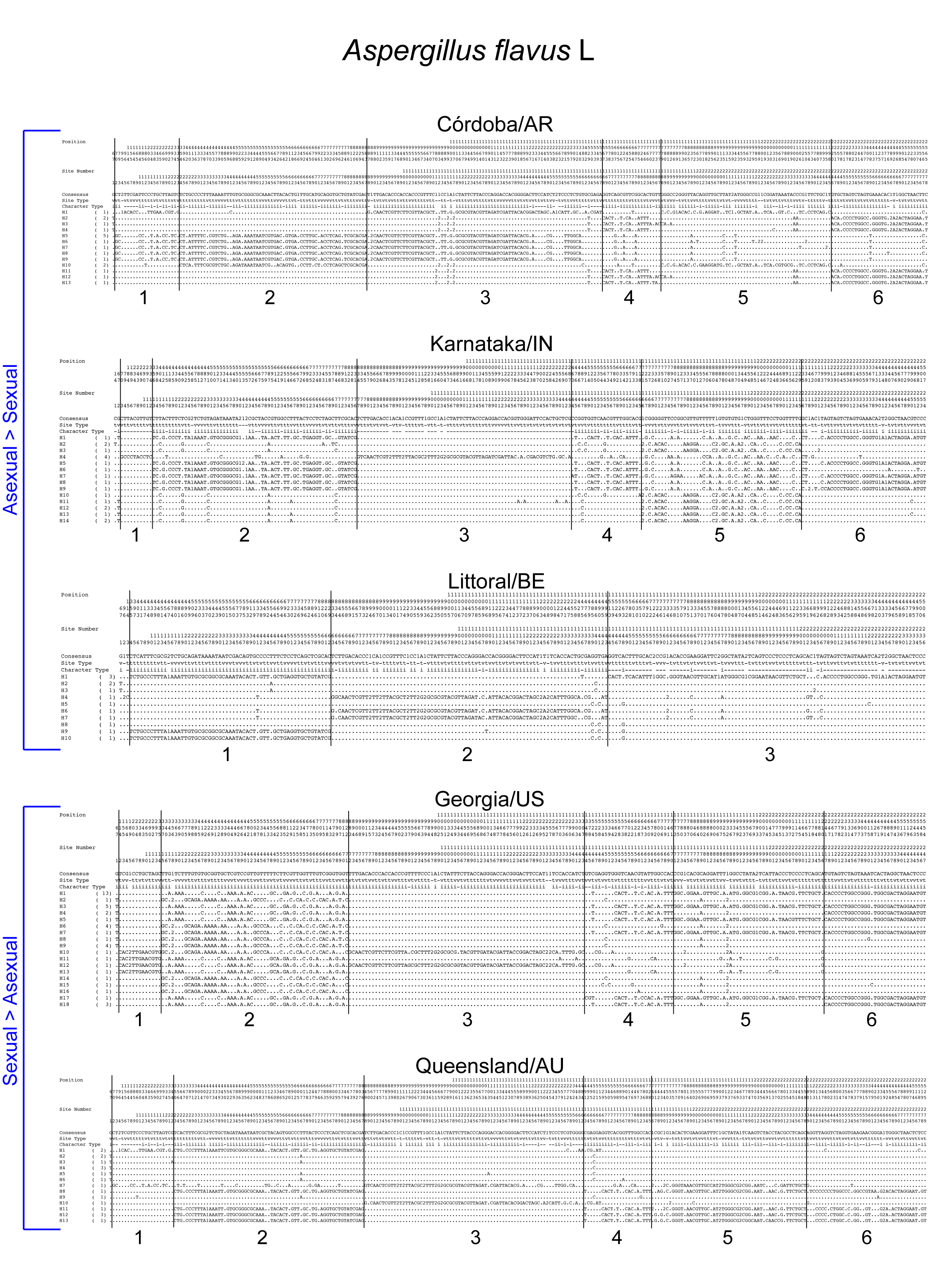

Supplement: Figure S1 — Distribution of SNPs and indels among haplotypes for each A. flavus L-strain population. Vertical lines correspond to putative boundaries for distinct LD blocks shown in Figure 3. (TIF) [file ppat.1003574.s001.tif]

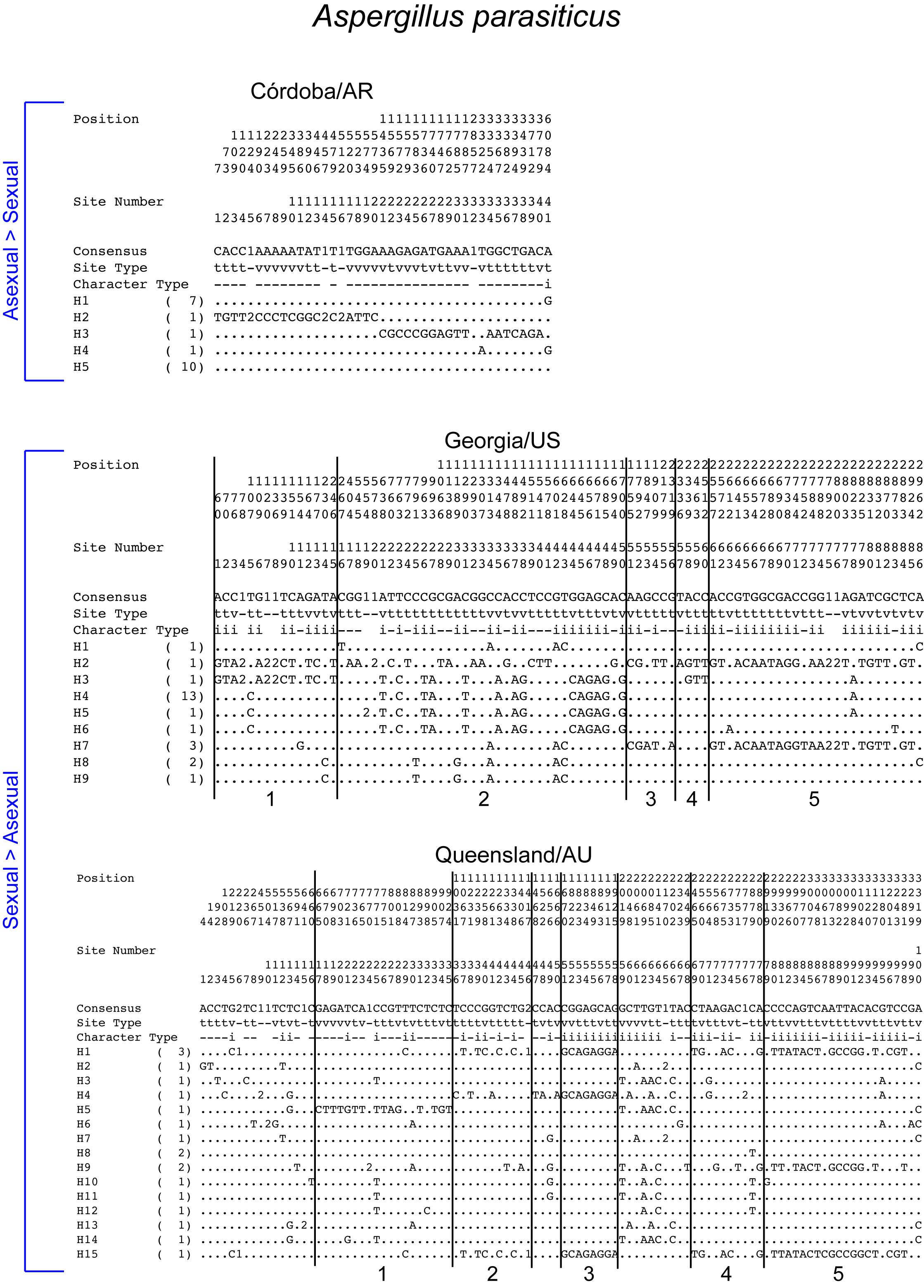

Supplement: Figure S2 — Distribution of SNPs and indels among haplotypes for each A. parasiticus population. Vertical lines correspond to putative boundaries for distinct LD blocks shown in Figure 3. There was no evidence of recombination in the Argentina population and the entire region examined falls into a single LD block. (TIF) [file ppat.1003574.s002.tif]

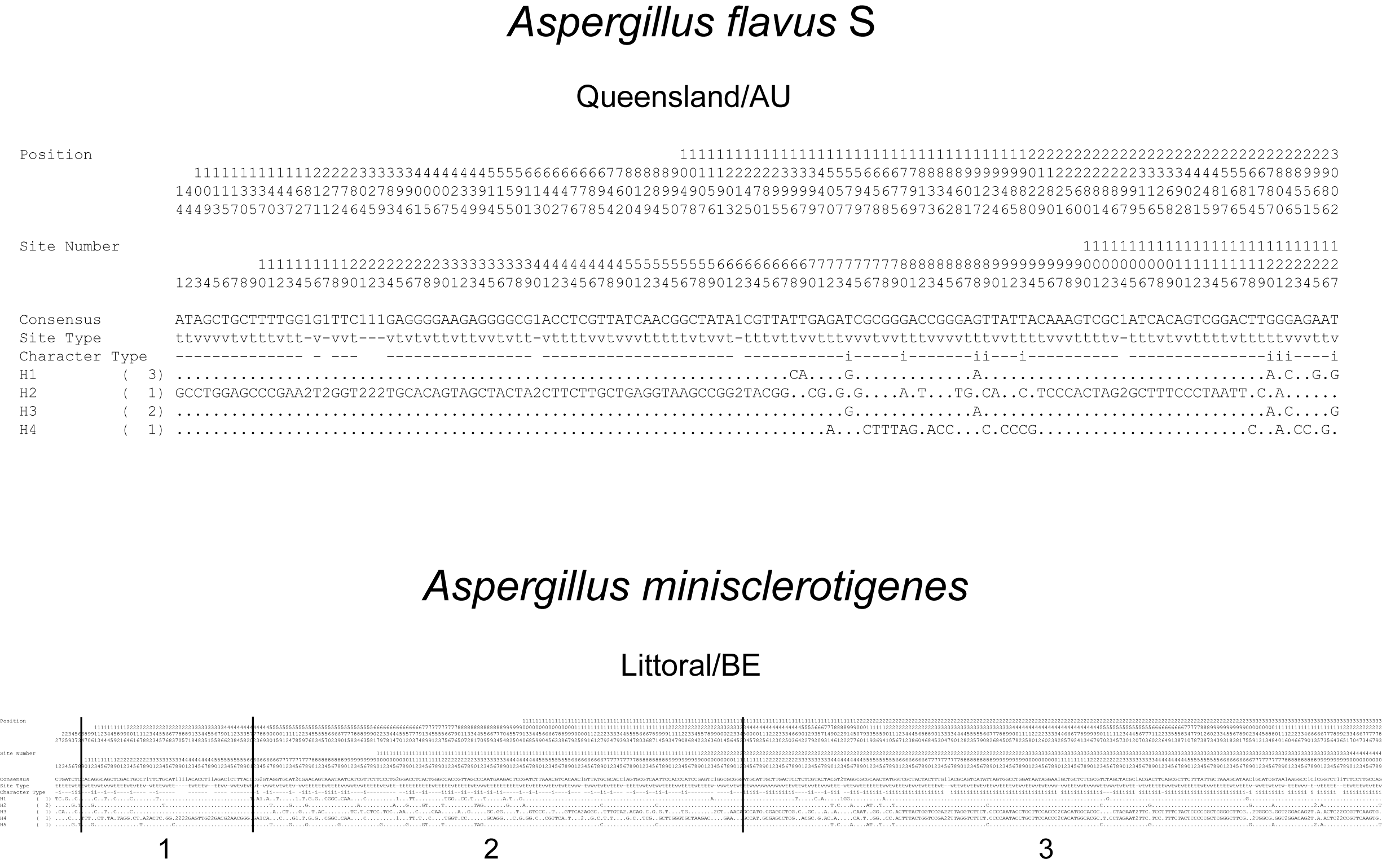

Supplement: Figure S3 — Distribution of SNPs and indels among haplotypes for each A. flavus S-strain and A. minisclerotigene population in Australia and Benin, respectively. Vertical lines correspond to putative boundaries for distinct LD blocks shown in Figure 3. There was no evidence of recombination in A. flavus S sampled in Australia. (TIF) [file ppat.1003574.s003.tif]
