# Supplementary material for: Sexuality Generates Diversity in the Aflatoxin Gene Cluster: Evidence on a Global Scale
Source: PLoS Pathog. 2013 Aug 29;9(8):e1003574. doi: 10.1371/journal.ppat.1003574 (PMC3757046; doi:10.1371/journal.ppat.1003574)
Supplement: Table S1 — Aspergillus flavus L isolates from Córdoba, Argentina. (DOC) [file ppat.1003574.s004.doc]

Table S1. *Aspergillus flavus* L isolates from Córdoba, Argentina.

| **IC Strain** | ***MAT*** | **B_1_ (μg/mL)^a^** | **B_2_ (μg/mL)^a^** | **Total B (μg/mL)** | **MLST^b^** |
| --- | --- | --- | --- | --- | --- |
| 396^c^ | 1 | 0.0 (0) | 0.0 (0) | 0.0 | H1 |
| 397^d^ | 1 | 0.0 (0) | 0.0 (0) | 0.0 | H2 |
| 398^d^ | 1 | 0.0 (0) | 0.0 (0) | 0.0 | H2 |
| 399 | 1 | 15.1 (1) | 0.2 (0) | 15.3 | H3 |
| 400 | 1 | 0.0 (0) | 0.0 (0) | 0.0 | H4 |
| 401^d^ | 1 | 0.0 (0) | 0.0 (0) | 0.0 | H2 |
| 402^d^ | 1 | 0.0 (0) | 0.0 (0) | 0.0 | H10 |
| 403 | 1 | 0.0 (0) | 0.0 (0) | 0.0 | H1 |
| 404^c,d^ | 1 | 0.0 (0) | 0.0 (0) | 0.0 | H2 |
| 405 | 2 | 0.0 (0) | 0.0 (0) | 0.0 | H5 |
| 406 | 1 | 62.3 (21) | 0.7 (0.3) | 63.0 | H6 |
| 407 | 1 | 0.0 (0) | 0.0 (0) | 0.0 | H4 |
| 408^c,d^ | 1 | 0.0 (0) | 0.0 (0) | 0.0 | H2 |
| 409 | 2 | 112.2 (17) | 2 (0.3) | 114.2 | H13 |
| 410 | 1 | 22.4 (0.8) | 0.1 (0) | 22.5 | H19 |
| 411 | 1 | 76.3 (9) | 1.3 (0.1) | 77.6 | H15 |
| 412^c^ | 1 | 0.0 (0) | 0.0 (0) | 0.0 | H4 |
| 413 | 1 | 43 (3) | 0.3 (0) | 43.3 | H17 |
| 414^d^ | 1 | 0.0 (0) | 0.0 (0) | 0.0 | H2 |
| 415 | 2 | 138.4 (31) | 2.8 (0.8) | 141.2 | H13 |
| 416^c^ | 1 | 70.8 (6) | 1.2 (0.1) | 72.0 | H15 |
| 417 | 2 | 14.5 (5) | 0.1 (0) | 14.6 | H20 |
| 418^d^ | 1 | 0.0 (0) | 0.0 (0) | 0.0 | H2 |
| 419^d^ | 1 | 0.0 (0) | 0.0 (0) | 0.0 | H2 |
| 420^c^ | 1 | 94.8 (19) | 1.8 (0.3) | 96.6 | H16 |
| 421 | 1 | 17.3 (4) | 0.1 (0) | 17.4 | H19 |
| 422 | 1 | 190.1 (22) | 5 (0.5) | 195.1 | H12 |
| 423^d^ | 1 | 0.0 (0) | 0.0 (0) | 0.0 | H2 |
| 424^c^ | 2 | 115.5 (29) | 2.4 (0.8) | 117.9 | H13 |
| 425^d^ | 1 | 0.0 (0) | 0.0 (0) | 0.0 | H10 |
| 426 | 1 | 72.2 (16) | 1.3 (0.2) | 73.5 | H15 |
| 427 | 2 | 10.1 (0.8) | 0.1 (0) | 10.2 | H20 |
| 428^c,d^ | 1 | 0.0 (0) | 0.0 (0) | 0.0 | H10 |
| 429 | 1 | 0.0 (0) | 0.0 (0) | 0.0 | H4 |
| 430 | 1 | 0.7 (0.2) | 0.0 (0) | 0.7 | H24 |
| 431^d^ | 1 | 0.0 (0) | 0.0 (0) | 0.0 | H2 |
| 432^c,d^ | 1 | 0.0 (0) | 0.0 (0) | 0.0 | H10 |
| 433^d^ | 1 | 0.0 (0) | 0.0 (0) | 0.0 | H10 |
| 434^d^ | 1 | 0.0 (0) | 0.0 (0) | 0.0 | H2 |
| 435^d^ | 1 | 0.0 (0) | 0.0 (0) | 0.0 | H10 |
| 436^c^ | 1 | 90.5 (9) | 1.6 (0.2) | 92.1 | H18 |
| 437^d^ | 1 | 0.0 (0) | 0.0 (0) | 0.0 | H10 |
| 438 | 1 | 3.6 (0.7) | 0.0 (0) | 3.6 | H1 |
| 439^d^ | 1 | 0.0 (0) | 0.0 (0) | 0.0 | H10 |
| 440^c^ | 2 | 15.7 (6) | 0.1 (0) | 15.8 | H21 |
| 441 | 1 | 0.0 (0) | 0.0 (0) | 0.0 | H1 |
| 442^d^ | 1 | 0.0 (0) | 0.0 (0) | 0.0 | H2 |
| 443 | 1 | 112.9 (24) | 1.8 (0.2) | 114.7 | H16 |
| 444^c^ | 1 | 0.0 (0) | 0.0 (0) | 0.0 | H4 |
| 445 | 1 | 1.2 (0.3) | 0.0 (0) | 1.2 | H2 |
| 446 | 1 | 76.7 (18) | 1.2 (0.3) | 77.9 | H15 |
| 447^d^ | 1 | 0.0 (0) | 0.0 (0) | 0.0 | H10 |
| 448^c,d^ | 1 | 0.0 (0) | 0.0 (0) | 0.0 | H10 |
| 449^d^ | 1 | 0.0 (0) | 0.0 (0) | 0.0 | H10 |
| 450 | 1 | 73.7 (9) | 1.2 (0.2) | 74.9 | H15 |
| 451 | 1 | 0.1 (0.1) | 0.0 (0) | 0.1 | H23 |
| 452^c,d^ | 1 | 0.0 (0) | 0.0 (0) | 0.0 | H2 |
| 453^d^ | 1 | 0.0 (0) | 0.0 (0) | 0.0 | H2 |
| 454^d^ | 1 | 0.1 (0) | 0.0 (0) | 0.1 | H22 |
| 455 | 1 | 0.0 (0) | 0.0 (0) | 0.0 | H24 |
| 456^c,d^ | 1 | 0.0 (0) | 0.0 (0) | 0.0 | H2 |
| 457 | 2 | 28.5 (5) | 0.1 (0) | 28.6 | H14 |
| 458^d^ | 1 | 0.0 (0) | 0.0 (0) | 0.0 | H8 |
| 459 | 2 | 21.8 (2) | 0.2 (0) | 22.0 | H17 |
| 460^c,d^ | 1 | 0.0 (0) | 0.0 (0) | 0.0 | H2 |
| 461^d^ | 1 | 0.0 (0) | 0.0 (0) | 0.0 | H2 |
| 462 | 1 | 35.7 (3) | 0.1 (0) | 35.8 | H7 |
| 463^d^ | 1 | 0.0 (0) | 0.0 (0) | 0.0 | H10 |
| 464^c,d^ | 1 | 0.0 (0) | 0.0 (0) | 0.0 | H10 |
| 465^d^ | 1 | 0.0 (0) | 0.0 (0) | 0.0 | H10 |
| 466^d^ | 1 | 0.0 (0) | 0.0 (0) | 0.0 | H11 |
| 467 | 1 | 0.0 (0) | 0.0 (0) | 0.0 | H9 |
| 468^c^ | 2 | 7.9 (0.9) | 0.1 (0) | 8.0 | H21 |
| 469^d^ | 1 | 0.0 (0) | 0.0 (0) | 0.0 | H2 |
| 470 | 2 | 11.6 (3) | 0.1 (0) | 11.7 | H20 |
| 471 | 2 | 10.2 (4) | 0.1 (0) | 10.3 | H20 |
| 472^c^ | 1 | 62.1 (4) | 0.9 (0) | 63.0 | H15 |
| 474 | 1 | 0.0 (0) | 0.0 (0) | 0.0 | H1 |
| 475 | 1 | 76.4 (10) | 1.1 (0.2) | 77.5 | H15 |

^a^ AF concentration is based on average of three replicate cultures per isolate.

Number in parentheses is standard deviation.

^b^ Haplotypes based on four genomic loci: *aflM/aflN*, *aflW/aflX*, *amdS*, *trpC*.

^c^ Isolate part of a subset for LD analysis in Figure 3.

^d^ AF- isolate groups with Geiser’s IB clade (25).
