# Supplementary material for: Sexuality Generates Diversity in the Aflatoxin Gene Cluster: Evidence on a Global Scale
Source: PLoS Pathog. 2013 Aug 29;9(8):e1003574. doi: 10.1371/journal.ppat.1003574 (PMC3757046; doi:10.1371/journal.ppat.1003574)
Supplement: Table S2 — Aspergillus flavus L isolates from Karnataka, India. (DOC) [file ppat.1003574.s005.doc]

Table S2. *Aspergillus flavus* L isolates from Karnataka, India.

| **IC Strain** | ***MAT*** | **B_1_ (μg/mL)^a^** | **B_2_ (μg/mL)^a^** | **Total B (μg/mL)** | **MLST^b^** |
| --- | --- | --- | --- | --- | --- |
| 1229^c^ | 2 | 20.2 (7) | 0.2 (0.1) | 20.4 | H1 |
| 1230^c^ | 2 | 116.1 (72) | 0.6 (0.4) | 116.7 | H2 |
| 1231 | 2 | 21.7 (3) | 0.5 (0.1) | 22.2 | - |
| 1232 | 2 | 125.9 (13) | 3.8 (0.4) | 129.7 | - |
| 1233 | 1 | 100.4 (12) | 0.7 (0.1) | 101.1 | H19 |
| 1234 | 1 | 0.3 (0) | 0.0 (0) | 0.30 | - |
| 1235 | 1 | 19 (2) | 0.4 (0) | 19.4 | - |
| 1236 | 1 | 21 (9) | 0.3 (0.2) | 21.3 | - |
| 1237 | 2 | 80.1 (8) | 3.0 (0.2) | 83.1 | H33 |
| 1238 | 1 | 64.1 (9) | 1.4 (0.4) | 65.5 | - |
| 1239^c^ | 1 | 0.0 (0) | 0.0 (0) | 0.0 | H9 |
| 1240 | 2 | 58.6 (27) | 0.8 (0.3) | 59.4 | - |
| 1241^c^ | 1 | 9.7 (3) | 0.1 (0) | 9.8 | H10 |
| 1242 | 2 | 70.3 (9) | 1.0 (0.1) | 71.3 | - |
| 1243 | 1 | 75.2 (6) | 0.9 (0.1) | 76.1 | - |
| 1244 | 1 | 45 (23) | 1.2 (0.8) | 46.2 | - |
| 1245^c^ | 2 | 66.4 (12) | 1.3 (0.1) | 67.7 | H25 |
| 1246 | 2 | 66.6 (7) | 1.4 (0.2) | 68.0 | - |
| 1247 | 2 | 0.0 (0) | 0.0 (0) | 0.0 | - |
| 1248 | 2 | 84.5 (5) | 1.3 (0.2) | 85.8 | - |
| 1249^c^ | 2 | 162.9 (26) | 1.1 (0.1) | 164 | H2 |
| 1250 | 1 | 58 (9) | 1.1 (0.2) | 59.1 | H26 |
| 1251 | 1 | 17.2 (6) | 0.1 (0) | 17.3 | H17 |
| 1252 | 1 | 13.4 (2) | 0.3 (0) | 13.7 | H28 |
| 1253^c^ | 1 | 34.7 (21) | 0.6 (0.4) | 35.3 | H27 |
| 1254 | 2 | 104.9 (6) | 1.5 (0.2) | 106.4 | H16 |
| 1255 | 1 | 35.7 (3) | 1.1 (0.2) | 36.8 | H30 |
| 1256 | 2 | 30.4 (9) | 0.6 (0.2) | 31.0 | - |
| 1257^c^ | 1 | 0.1 (0) | 0.0 (0) | 0.10 | H12 |
| 1258 | 1 | 10.6 (0.8) | 0.1 (0) | 10.7 | H18 |
| 1259 | 1 | 33.3 (5) | 1.1 (0.2) | 34.4 | - |
| 1260 | 2 | 112.5 (11) | 0.6 (0.1) | 113.1 | H2 |
| 1261 | 1 | 0.0 (0) | 0.0 (0) | 0.0 | - |
| 1262 | 1 | 56.1 (12) | 0.5 (0.1) | 56.6 | H21 |
| 1263 | 2 | 15 (2) | 0.2 (0) | 15.2 | - |
| 1264 | 2 | 15.6 (5) | 0.1 (0) | 15.7 | H20 |
| 1265^c^ | 1 | 6.2 (2) | 0.0 (0) | 6.20 | H22 |
| 1266 | 1 | 13.4 (3) | 0.1 (0) | 13.5 | H3 |
| 1267 | 1 | 83.3 (7) | 3.0 (0.3) | 86.3 | - |
| 1268 | 1 | 63.4 (11) | 2.5 (0.4) | 65.9 | H31 |
| 1269^c^ | 1 | 0.1 (0.1) | 0.0 (0) | 0.10 | H34 |
| 1270 | 2 | 20.1 (12) | 0.9 (0.5) | 21.0 | H24 |
| 1271 | 1 | 3.2 (2) | 0.0 (0) | 3.20 | H14 |
| 1272 | 1 | 0.1 (0) | 0.0 (0) | 0.10 | H7 |
| 1273 | 1 | 49.4 (18) | 0.9 (0.5) | 50.3 | - |
| 1274^c,d^ | 2 | 0.0 (0) | 0.0 (0) | 0.0 | H4 |
| 1275 | 1 | 47 (9) | 0.2 (0) | 47.2 | H20 |
| 1276 | 1 | 46.2 (14) | 1.6 (0.5) | 47.8 | H32 |
| 1277^c^ | 1 | 18 (6) | 0.1 (0) | 18.1 | H23 |
| 1278 | 1 | 21.2 (2) | 0.6 (0.1) | 21.8 | - |
| 1279 | 2 | 49 (18) | 0.6 (0.2) | 49.6 | H13 |
| 1280^c^ | 1 | 105 (18) | 1.5 (0.2) | 106.5 | H11 |
| 1281 | 1 | 52.8 (41) | 0.5 (0.4) | 53.3 | H22 |
| 1282 | 1 | 0.4 (0.2) | 0.0 (0) | 0.40 | H12 |
| 1283 | 1 | 41.4 (23) | 0.3 (0.2) | 41.7 | - |
| 1284 | 2 | 38 (10) | 0.7 (0.2) | 38.7 | - |
| 1285 | 2 | 41.1 (9) | 0.7 (0.1) | 41.8 | - |
| 1286 | 1 | 31.5 (8) | 0.4 (0.1) | 31.9 | - |
| 1287 | 1 | 76.8 (26) | 0.8 (0.3) | 77.6 | - |
| 1288 | 1 | 4.0 (0.5) | 0.1 (0) | 4.10 | - |
| 1289 | 2 | 17.4 (8) | 0.1 (0.1) | 17.5 | - |
| 1290^c^ | 1 | 88.7 (32) | 0.8 (0.3) | 89.5 | H12 |
| 1291^c,d^ | 1 | 0.0 (0) | 0.0 (0) | 0.0 | H5 |
| 1292 | 1 | 53.1 (13) | 0.6 (0.1) | 53.7 | - |
| 1293^c,d^ | 2 | 0.0 (0) | 0.0 (0) | 0.0 | H4 |
| 1294 | 2 | 35.1 (14) | 0.8 (0.3) | 35.9 | - |
| 1295 | 2 | 43.9 (23) | 0.8 (0.6) | 44.7 | H29 |
| 1296 | 1 | 0.2 (0.1) | 0.0 (0) | 0.20 | H12 |
| 1297^c^ | 1 | 25.1 (9) | 0.5 (0.2) | 25.6 | H27 |
| 1298 | 1 | 14.2 (5) | 0.3 (0.1) | 14.5 | - |
| 1299 | 1 | 44.9 (16) | 0.9 (0.3) | 45.8 | - |
| 1300 | 2 | 30 (4) | 0.6 (0) | 30.6 | - |
| 1301 | 2 | 7.3 (4) | 0.0 (0) | 7.30 | - |
| 1302 | 1 | 14 (4) | 0.3 (0.1) | 14.3 | - |
| 1303^c,d^ | 1 | 0.0 (0) | 0.0 (0) | 0.0 | H6 |
| 1304^c^ | 1 | 0.0 (0) | 0.0 (0) | 0.0 | H9 |
| 1305 | 2 | 67.5 (15) | 0.9 (0.3) | 68.4 | H15 |
| 1306 | 1 | 29.6 (6) | 0.7 (0.1) | 30.3 | H8 |
| 1307^c^ | 1 | 73.1 (15) | 1.8 (0.4) | 74.9 | H1 |
| 1308 | 2 | 13.8 (2) | 0.3 (0.1) | 14.1 | - |

^a^ AF concentration is based on average of three replicate cultures per isolate.

Number in parentheses is standard deviation.

^b^ Haplotypes based on four genomic loci: *aflM/aflN*, *aflW/aflX*, *amdS*, *trpC*.

^c^ Isolate part of a subset for LD analysis in Figure 3.

^d^ AF- isolate groups with Geiser’s IB clade (25).
