# Supplementary material for: Sexuality Generates Diversity in the Aflatoxin Gene Cluster: Evidence on a Global Scale
Source: PLoS Pathog. 2013 Aug 29;9(8):e1003574. doi: 10.1371/journal.ppat.1003574 (PMC3757046; doi:10.1371/journal.ppat.1003574)
Supplement: Table S3 — Aspergillus flavus L isolates from Littoral, Benin. (DOC) [file ppat.1003574.s006.doc]

Table S3. *Aspergillus flavus* L isolates from Littoral, Benin.

| **IC Strain** | ***MAT*** | **B_1_ (μg/mL)^a^** | **B_2_ (μg/mL)^a^** | **Total B (μg/mL)** | **MLST^b^** |
| --- | --- | --- | --- | --- | --- |
| 1027^c^ | 1 | 83 (52) | 2.3 (1) | 85.3 | H1 |
| 1028 | 1 | 59.6 (25) | 0.5 (0.2) | 60.1 | H2 |
| 1029 | 2 | 46 (5) | 1.2 (0) | 47.2 | H50 |
| 1030 | 1 | 86 (14) | 1.8 (0.4) | 87.8 | H54 |
| 1031 | 2 | 88.5 (47) | 4.6 (1) | 93.1 | H59 |
| 1032 | 1 | 42.4 (19) | 3.2 (0.5) | 45.6 | H60 |
| 1033 | 2 | 152.8 (13) | 6.7 (1) | 159.5 | H16 |
| 1034 | 2 | 53.3 (11) | 1.4 (0.3) | 54.7 | H51 |
| 1035^c^ | 1 | 52.8 (11) | 0.9 (0.1) | 53.7 | H32 |
| 1036 | 2 | 28.8 (18) | 0.5 (0.2) | 29.3 | H34 |
| 1037 | 1 | 45.4 (12) | 0.8 (0.3) | 46.2 | H17 |
| 1038 | 2 | 22.4 (8) | 0.4 (0.1) | 22.8 | H28 |
| 1039 | 2 | 0.0 (0) | 0.0 (0) | 0.0 | H6 |
| 1040 | 2 | 81.5 (37) | 2.2 (1) | 83.7 | H56 |
| 1041 | 1 | 63.8 (16) | 0.8 (0.1) | 64.6 | H17 |
| 1042 | 2 | 0.0 (0) | 0.0 (0) | 0.0 | H38 |
| 1043^c^ | 1 | 0.0 (0) | 0.0 (0) | 0.0 | H10 |
| 1044 | 1 | 0.0 (0) | 0.0 (0) | 0.0 | H25 |
| 1045 | 1 | 0.0 (0) | 0.0 (0) | 0.0 | H8 |
| 1046 | 2 | 13.5 (5) | 0.8 (0.2) | 14.3 | H1 |
| 1047 | 2 | 82.3 (18) | 2 (0.6) | 84.3 | H58 |
| 1048 | 1 | 0.0 (0) | 0.0 (0) | 0.0 | H25 |
| 1049 | 1 | 79.4 (7) | 1.9 (0.1) | 81.3 | H26 |
| 1050 | 1 | 8.7 (4) | 0.3 (0) | 9.00 | H5 |
| 1051^c^ | 2 | 11.7 (2) | 0.5 (0.1) | 12.2 | H1 |
| 1052 | 2 | 85.5 (15) | 0.5 (0.1) | 86.0 | H19 |
| 1053 | 2 | 0.0 (0) | 0.0 (0) | 0.0 | H6 |
| 1054^d^ | 2 | 0.0 (0) | 0.0 (0) | 0.0 | H39 |
| 1055 | 1 | 0.0 (0) | 0.0 (0) | 0.0 | H13 |
| 1056 | 1 | 21.5 (4) | 0.1 (0) | 21.6 | H37 |
| 1057 | 2 | 17.9 (1) | 0.5 (0.1) | 18.4 | H55 |
| 1058 | 2 | 13.2 (3) | 0.1 (0) | 13.3 | H47 |
| 1059^c^ | 1 | 102.4 (13) | 1.2 (0.1) | 103.6 | H17 |
| 1060 | 1 | 61.8 (10) | 0.4 (0) | 62.2 | H20 |
| 1061^d^ | 1 | 0.0 (0) | 0.0 (0) | 0.0 | H3 |
| 1062 | 1 | 235 (83) | 10.1 (5) | 245.1 | H57 |
| 1063 | 1 | 0.0 (0) | 0.0 (0) | 0.0 | H36 |
| 1064 | 1 | 153.2 (20) | 1.8 (0.4) | 155.0 | H33 |
| 1065 | 2 | 4.7 (1) | 0.0 (0) | 4.80 | H2 |
| 1066 | 1 | 69.9 (16) | 1.5 (0.6) | 71.4 | H29 |
| 1067^c^ | 1 | 0.0 (0) | 0.0 (0) | 0.0 | H15 |
| 1068 | 1 | 0.0 (0) | 0.0 (0) | 0.0 | H6 |
| 1069 | 2 | 20.4 (5) | 0.3 (0) | 20.7 | H51 |
| 1070 | 1 | 0.0 (0) | 0.0 (0) | 0.0 | H6 |
| 1071 | 1 | 0.0 (0) | 0.0 (0) | 0.0 | H12 |
| 1072 | 2 | 55.7 (13) | 1.0 (0.2) | 56.7 | H2 |
| 1073 | 2 | 54.4 (36) | 0.8 (0.4) | 55.2 | H2 |
| 1074 | 2 | 124 (23) | 2.6 (0.4) | 126.6 | H4 |
| 1075^c^ | 1 | 126.7 (102) | 5.1 (3) | 131.8 | H52 |
| 1076 | 1 | 87.3 (37) | 2.2 (0.7) | 89.5 | H53 |
| 1077 | 1 | 113.7 (24) | 4.6 (0.6) | 118.3 | H42 |
| 1078 | 1 | 106.6 (40) | 2.8 (1) | 109.4 | H40 |
| 1079 | 2 | 130.5 (67) | 1.8 (0.9) | 132.3 | H22 |
| 1080 | 1 | 38.5 (12) | 3 (0.3) | 41.5 | H41 |
| 1081 | 1 | 165.5 (49) | 5.7 (1) | 171.2 | H17 |
| 1082 | 1 | 0.0 (0) | 0.0 (0) | 0.0 | H27 |
| 1083^c^ | 2 | 40 (6) | 0.9 (0.2) | 40.9 | H14 |
| 1084 | 1 | 1.3 (0.3) | 0.0 (0) | 1.30 | H9 |
| 1085 | 1 | 1.2 (0.2) | 0.0 (0) | 1.20 | H11 |
| 1086 | 1 | 237.3 (29) | 9.6 (0.9) | 246.9 | H45 |
| 1087 | 1 | 25.8 (10) | 0.2 (0.1) | 26.0 | H30 |
| 1088 | 1 | 126.5 (28) | 4.9 (0.9) | 131.4 | H43 |
| 1089 | 2 | 0.0 (0) | 0.0 (0) | 0.0 | H2 |
| 1090 | 2 | 76.4 (12) | 1.5 (0.3) | 77.9 | H23 |
| 1091^c^ | 1 | 0.2 (0.1) | 0.0 (0) | 0.2 | H38 |
| 1092 | 1 | 157.7 (31) | 6.4 (1) | 164.1 | H44 |
| 1093 | 1 | 26.9 (4) | 0.2 (0) | 27.1 | H34 |
| 1094 | 1 | 0.0 (0) | 0.0 (0) | 0.0 | H31 |
| 1095 | 2 | 121.9 (8) | 2.2 (0.3) | 124.1 | H48 |
| 1096 | 1 | 54.5 (24) | 0.6 (0.3) | 55.1 | H24 |
| 1097^c^ | 2 | 285.5 (57) | 7.1 (2) | 292.6 | H18 |
| 1098 | 1 | 192.1 (67) | 6.4 (2) | 198.5 | H21 |
| 1099^c^ | 2 | 0.0 (0) | 0.0 (0) | 0.0 | H38 |
| 1100^c,e^ | 2 | 0.1 (0) | 26.2 (7) | 26.3 | H49 |
| 1101 | 1 | 0.0 (0) | 0.0 (0) | 0.0 | H34 |
| 1102 ^c,e^ | 2 | 0.1 (0) | 24.3 (3) | 24.4 | H49 |
| 1103 | 1 | 62.2 (5) | 1.4 (0.2) | 63.6 | H35 |
| 1104 | 1 | 79.3 (16) | 1.8 (0.4) | 81.1 | H35 |
| 1105 | 1 | 157.9 (27) | 5.6 (0.6) | 163.5 | H46 |
| 1106 | 1 | 1.6 (0.6) | 0.0 (0) | 1.60 | H7 |

^a^ AF concentration is based on average of three replicate cultures per isolate.

Number in parentheses is standard deviation.

^b^ Haplotypes based on four genomic loci: *aflM/aflN*, *aflW/aflX*, *amdS*, *trpC*.

^c^ Isolate part of a subset for LD analysis in Figure 3.

^d^ AF- isolate groups with Geiser’s IB clade (25).

^e^ Isolate produces more B_2_ than B_1_.
