# Supplementary material for: Sexuality Generates Diversity in the Aflatoxin Gene Cluster: Evidence on a Global Scale
Source: PLoS Pathog. 2013 Aug 29;9(8):e1003574. doi: 10.1371/journal.ppat.1003574 (PMC3757046; doi:10.1371/journal.ppat.1003574)
Supplement: Table S4 — Aspergillus flavus L isolates from Georgia, United States. (DOC) [file ppat.1003574.s007.doc]

Table S4. *Aspergillus flavus* L isolates from Georgia, United States.

| **IC Strain** | ***MAT*** | **B_1_ (μg/mL)^a^** | **B_2_ (μg/mL)^a^** | **Total B (μg/mL)** | **MLST^b^** |
| --- | --- | --- | --- | --- | --- |
| 229^c^ | 2 | 39.2 (22) | 0.8 (0.5) | 40.0 | H1 |
| 230 | 2 | 15.8 (13) | 0.3 (0.2) | 16.1 | H1 |
| 231 | 2 | 33.7 (13) | 0.7 (0.2) | 34.4 | H1 |
| 232 | 2 | 16.6 (11) | 0.4 (0.2) | 17.0 | H1 |
| 233 | 2 | 31.4 (7) | 0.7 (0.1) | 32.1 | H1 |
| 234^c^ | 2 | 85.6 (6) | 1.4 (0.3) | 87.0 | H2 |
| 235 | 2 | 104 (17) | 1.8 (0.1) | 105.8 | H2 |
| 236 | 2 | 106 (15) | 1.6 (0.7) | 107.6 | H2 |
| 237 | 2 | 130.4 (13) | 2.5 (0.9) | 132.9 | H2 |
| 238 | 2 | 98.6 (18) | 1.9 (0.6) | 100.5 | H2 |
| 239 | 2 | 86.7 (24) | 1.4 (0.4) | 88.1 | H2 |
| 240 | 2 | 101.2 (20) | 1.9 (0.3) | 103.1 | H2 |
| 241 | 2 | 59.5 (15) | 0.9 (0.7) | 60.4 | H2 |
| 242 | 2 | 115.1 (11) | 1.4 (0.5) | 116.5 | H2 |
| 244^c^ | 1 | 104.3 (10) | 1.7 (0.1) | 106.0 | H6 |
| 245^c^ | 2 | 96.3 (6) | 1.9 (0.6) | 98.2 | H28 |
| 246 | 2 | 100 (9) | 2.2 (0.1) | 102.2 | H28 |
| 247 | 2 | 94.2 (13) | 2.0 (0.3) | 96.2 | H28 |
| 248 | 2 | 131.8 (5) | 2.8 (0.6) | 134.6 | H28 |
| 249 | 2 | 119.8 (13) | 3.1 (0.5) | 122.9 | H28 |
| 250 | 2 | 120.1 (17) | 3.1 (0.5) | 123.2 | H28 |
| 251 | 2 | 157 (20) | 3.8 (0.7) | 160.8 | H28 |
| 252 | 2 | 0.0 (0) | 0.0 (0) | 0.0 | - |
| 253 | 2 | 0.0 (0) | 0.0 (0) | 0.0 | H29 |
| 254 | 2 | 0.0 (0) | 0.0 (0) | 0.0 | - |
| 255 | 2 | 0.0 (0) | 0.0 (0) | 0.0 | - |
| 256 | 2 | 0.0 (0) | 0.0 (0) | 0.0 | - |
| 257 | 2 | 0.0 (0) | 0.0 (0) | 0.0 | - |
| 258^c^ | 2 | 168.3 (29) | 4.4 (0.6) | 172.7 | H7 |
| 259 | 2 | 134.3 (5) | 3.8 (0.8) | 138.1 | H7 |
| 260 | 2 | 210.6 (36) | 6 (0.8) | 216.6 | H7 |
| 261 | 2 | 193.5 (19) | 5.6 (0.9) | 199.1 | H7 |
| 262 | 2 | 213 (22) | 6.1 (1) | 219.1 | H7 |
| 263^c^ | 2 | 0.8 (0.1) | 0.0 (0) | 0.8 | H30 |
| 264 | 2 | 0.5 (0.1) | 0.0 (0) | 0.5 | H30 |
| 265 | 2 | 0.1 (0.1) | 0.0 (0) | 0.1 | H30 |
| 267^c^ | 2 | 109 (41) | 2.5 (1) | 111.5 | H34 |
| 268 | 2 | 141.2 (50) | 3.8 (2) | 145.0 | H34 |
| 269 | 2 | 79 (37) | 2.5 (1) | 81.5 | H34 |
| 270^c^ | 2 | 72.5 (7) | 3.3 (0.7) | 75.8 | H17 |
| 271 | 2 | 89.4 (7) | 2.1 (1) | 91.5 | H17 |
| 272^c^ | 2 | 64.2 (7) | 1.2 (0.2) | 65.4 | H22 |
| 273 | 2 | 53.7 (11) | 0.4 (0.1) | 54.1 | H22 |
| 274^c^ | 2 | 4.8 (2) | 0.1 (0.1) | 4.9 | H12 |
| 275 | 2 | 2.4 (1) | 0.1 (0) | 2.5 | H12 |
| 276^c^ | 2 | 0.4 (0.1) | 0.0 (0) | 0.4 | H24 |
| 277^c,d^ | 2 | 0.0 (0) | 0.0 (0) | 0.0 | H3 |
| 278^c^ | 1 | 99.4 (46) | 1.8 (1) | 101.2 | H9 |
| 279^c^ | 2 | 12.9 (1) | 0.1 (0) | 13.0 | H37 |
| 280^c^ | 2 | 36.9 (8) | 1.3 (0.5) | 38.2 | H21 |
| 281^c^ | 2 | 154 (9) | 2.6 (0.5) | 156.6 | H18 |
| 282^c^ | 1 | 164.6 (27) | 5.5 (1) | 170.1 | H14 |
| 283^c^ | 1 | 9.6 (1) | 0.1 (0) | 9.7 | H32 |
| 284^c^ | 2 | 24.6 (3) | 0.4 (0) | 25.0 | H23 |
| 285^c^ | 2 | 185.3 (47) | 1.5 (0.8) | 186.8 | H31 |
| 286^c^ | 2 | 24.7 (4) | 0.7 (0.1) | 25.4 | H1 |
| 287^c^ | 2 | 104.1 (28) | 2.3 (1) | 106.4 | H8 |
| 288^c^ | 2 | 97.4 (18) | 1.8 (0.6) | 99.2 | H35 |
| 289^c^ | 1 | 40.2 (26) | 0.7 (0.4) | 40.9 | H26 |
| 290^c^ | 2 | 15.3 (5) | 0.3 (0.1) | 15.6 | H2 |
| 291^c^ | 1 | 47.6 (14) | 0.3 (0.1) | 47.9 | H27 |
| 292^c^ | 2 | 105.6 (25) | 2.8 (0.4) | 108.4 | H11 |
| 293^c^ | 1 | 54.2 (13) | 1 (0.2) | 55.2 | H21 |
| 294^c^ | 2 | 19.6 (5) | 0.1 (0) | 19.7 | H2 |
| 295^c^ | 2 | 164.6 (17) | 4.7 (0.8) | 169.3 | H13 |
| 296^c^ | 1 | 0.3 (0.1) | 0.0 (0) | 0.3 | H25 |
| 297^c^ | 1 | 25.7 (11) | 0.8 (0.4) | 26.5 | H15 |
| 298^c^ | 1 | 18.7 (4) | 0.2 (0) | 18.9 | H19 |
| 299^c^ | 2 | 36.2 (19) | 0.8 (0.5) | 37.0 | H22 |
| 300^c^ | 1 | 51.4 (11) | 0.6 (0.2) | 52.0 | H2 |
| 301^c^ | 1 | 16.8 (3) | 0.1 (0) | 16.9 | H16 |
| 302^c^ | 2 | 83.2 (16) | 1.7 (0.6) | 84.9 | H20 |
| 303^c^ | 1 | 94.9 (15) | 1.8 (0.2) | 96.7 | H10 |
| 304^c^ | 2 | 69.6 (12) | 1.5 (0.7) | 71.1 | H11 |
| 305^c^ | 1 | 37.4 (11) | 0.5 (0.2) | 37.9 | H33 |
| 306^c^ | 2 | 11.5 (0.7) | 0.2 (0.1) | 11.7 | H36 |
| 307^c^ | 2 | 139.8 (5) | 3.1 (0.1) | 142.9 | H4 |
| 308^c^ | 1 | 39.8 (7) | 0.6 (0.1) | 40.4 | H5 |

^a^ AF concentration is based on average of three replicate cultures per isolate.

Number in parentheses is standard deviation.

^b^ Haplotypes based on four genomic loci: *aflM/aflN*, *aflW/aflX*, *amdS*, *trpC*.

^c^ Isolate part of a subset for LD analysis in Figure 3.

^d^ AF- isolate groups with Geiser’s IB clade (25).
