# Supplementary material for: Sexuality Generates Diversity in the Aflatoxin Gene Cluster: Evidence on a Global Scale
Source: PLoS Pathog. 2013 Aug 29;9(8):e1003574. doi: 10.1371/journal.ppat.1003574 (PMC3757046; doi:10.1371/journal.ppat.1003574)
Supplement: Table S5 — Aspergillus flavus L isolates from Queensland, Australia. (DOC) [file ppat.1003574.s008.doc]

Table S5. *Aspergillus flavus* L isolates from Queensland, Australia.

| **IC Strain** | ***MAT*** | **B_1_ (μg/mL)^a^** | **B_2_ (μg/mL)^a^** | **Total B (μg/mL)** | **MLST^b^** |
| --- | --- | --- | --- | --- | --- |
| 640^c,e^ | 2 | 496.2 (368) | 7.5 (6) | 503.7 | H1 |
| 641 | 1 | 155 (23) | 2.4 (0.3) | 157.8 | - |
| 642^e^ | 2 | 162.6 (14) | 2.5 (0.1) | 165.1 | H28 |
| 643^c^ | 2 | 0.1 (0) | 0.0 (0) | 0.10 | H3 |
| 644 | 1 | 59.9 (6) | 1.0 (0.1) | 60.9 | - |
| 645 | 1 | 64.2 (6) | 1.0 (0.1) | 65.2 | - |
| 646 | 2 | 64.5 (19) | 1.1 (0.3) | 65.6 | H17 |
| 647 | 2 | 51.4 (19) | 0.9 (0.2) | 52.3 | - |
| 648^c^ | 1 | 55.2 (9) | 0.2 (0.1) | 55.4 | H5 |
| 649 | 2 | 119 (20) | 2.4 (0.6) | 121.4 | - |
| 650 | 1 | 13 (2) | 0.0 (0) | 13.0 | H10 |
| 651 | 2 | 112.5 (8) | 1.5 (0.2) | 114.0 | H29 |
| 652^e^ | 1 | 101.9 (19) | 2.8 (0.3) | 104.7 | H25 |
| 653 | 2 | 6.6 (1) | 0.1 (0) | 6.70 | - |
| 654^e^ | 2 | 72.8 (6) | 0.4 (0.1) | 73.2 | - |
| 655^c^ | 2 | 84.1 (22) | 1.4 (0.5) | 85.5 | H17 |
| 656^c^ | 2 | 147.2 (21) | 2.2 (0.5) | 149.4 | H26 |
| 657 | 2 | 42.7 (10) | 0.5 (0.2) | 43.2 | H23 |
| 658 | 2 | 149.1 (24) | 2.4 (0.7) | 151.5 | H26 |
| 659^c^ | 2 | 0.0 (0) | 0.0 (0) | 0.0 | H21 |
| 660 | 2 | 146.3 (32) | 2.1 (0.6) | 148.4 | H29 |
| 661 | 2 | 0.4 (0.1) | 0.0 (0) | 0.40 | H15 |
| 662^c^ | 1 | 99.5 (12) | 1.1 (0.1) | 100.6 | H11 |
| 663 | 2 | 0.3 (0) | 0.0 (0) | 0.30 | H15 |
| 664^c^ | 2 | 27.2 (4) | 0.1 (0) | 27.3 | H7 |
| 665 | 2 | 31.2 (7) | 0.1 (0) | 31.3 | - |
| 666 | 2 | 32.3 (4) | 0.1 (0) | 32.4 | H8 |
| 667 | 2 | 27.8 (12) | 0.1 (0) | 27.9 | H8 |
| 668 | 1 | 60.7 (14) | 0.6 (0.1) | 61.3 | - |
| 669 | 2 | 114 (9) | 1.8 (0.2) | 115.8 | - |
| 670 | 1 | 43.8 (13) | 0.2 (0.1) | 44.0 | H9 |
| 671^c^ | 1 | 0.0 (0) | 0.0 (0) | 0.0 | H6 |
| 672^c^ | 2 | 200.7 (161) | 3.9 (4) | 204.6 | H31 |
| 673^c,d^ | 2 | 0.0 (0) | 0.0 (0) | 0.0 | H4 |
| 674 | 2 | 71 (25) | 1.7 (0.5) | 72.7 | H24 |
| 675 | 1 | 0.2 (0) | 0.0 (0) | 0.20 | H27 |
| 676 | 2 | 124.3 (16) | 1.5 (0.2) | 125.8 | H29 |
| 677 | 2 | 53.8 (5) | 0.9 (0.2) | 54.7 | H12 |
| 678 | 2 | 42.2 (14) | 0.7 (0.2) | 42.9 | H18 |
| 679^c^ | 2 | 66.1 (16) | 0.6 (0.2) | 66.7 | H16 |
| 680^c^ | 2 | 88.8 (17) | 1.6 (0.4) | 90.4 | H18 |
| 681 | 2 | 105 (2) | 1.9 (0.2) | 106.9 | - |
| 682 | 1 | 96.7 (30) | 1.6 (0.7) | 98.3 | H18 |
| 683 | 2 | 141.4 (16) | 1.8 (0) | 143.2 | H30 |
| 684 | 1 | 90 (27) | 1.5 (0.6) | 91.5 | H32 |
| 685 | 1 | 117.2 (34) | 1.6 (0.5) | 118.8 | H20 |
| 686 | 2 | 0.0 (0) | 0.0 (0) | 0.0 | H14 |
| 687 | 2 | 116 (3) | 1.3 (0.1) | 117.3 | - |
| 688^c^ | 1 | 122 (11) | 1.8 (0.1) | 123.8 | H20 |
| 689 | 1 | 95.4 (7) | 0.7 (0) | 96.1 | - |
| 690 | 2 | 32.6 (4) | 0.2 (0) | 32.8 | - |
| 691 | 2 | 66 (11) | 0.3 (0.1) | 66.3 | - |
| 692 | 1 | 61.1 (22) | 1.0 (0.4) | 62.1 | - |
| 693 | 1 | 122 (21) | 3.4 (0.4) | 125.4 | - |
| 694 | 1 | 69.8 (7) | 1.1 (0.1) | 70.9 | - |
| 695 | 1 | 0.1 (0) | 0.0 (0) | 0.10 | H22 |
| 696^c^ | 2 | 0.0 (0) | 0.0 (0) | 0.0 | H14 |
| 697 | 2 | 135.8 (18) | 1.9 (0.5) | 137.7 | H29 |
| 698^c^ | 2 | 0.0 (0) | 0.0 (0) | 0.0 | H13 |
| 699 | 2 | 119 (39) | 1.4 (0.6) | 120.3 | - |
| 700 | 2 | 129 (29) | 1.6 (0.3) | 130.6 | - |
| 701 | 2 | 34 (7) | 0.4 (0.1) | 34.4 | H25 |
| 702 | 1 | 123.2 (34) | 2 (0.6) | 125.2 | H20 |
| 703 | 2 | 116.6 (19) | 1.4 (0.4) | 118.0 | H29 |
| 704^c^ | 1 | 139.3 (33) | 2.2 (0.6) | 141.5 | H19 |
| 705 | 1 | 119 (29) | 1.0 (0.3) | 120.0 | - |
| 706 | 2 | 113 (16) | 1.0 (0.1) | 114.0 | - |
| 707 | 2 | 39.2 (15) | 0.1 (0) | 39.3 | - |
| 708 | 1 | 89.7 (17) | 2 (0.4) | 91.7 | H21 |
| 709^f,g^ | 1 | 4.1 (0.9) | 23.2 (5) | 27.3 | H5 |
| 710 | 2 | 99.2 (29) | 2 (0.6) | 101.2 | - |
| 711 | 2 | 80.7 (14) | 0.5 (0.1) | 81.2 | H12 |
| 712^c^ | 1 | 67.2 (17) | 0.2 (0.1) | 67.4 | H5 |
| 713 | 2 | 109 (48) | 1.2 (0.7) | 110.2 | - |
| 714 | 1 | 86.1 (14) | 0.9 (0.1) | 87.0 | - |
| 715 | 2 | 86.7 (27) | 1.1 (0.5) | 87.8 | - |
| 716 | 2 | 95.1 (29) | 1.7 (0.6) | 96.8 | - |
| 717 | 1 | 87.7 (35) | 1.1 (0.6) | 88.8 | - |
| 718 | 1 | 51.6 (11) | 0.5 (0.1) | 52.1 | - |
| 719^c,f^ | 2 | 1.3 (0.5) | 0.0 (0) | 1.3 | H2 |

^a^ AF concentration is based on average of three replicate cultures per isolate.

Number in parentheses is standard deviation.

^b^ Haplotypes based on four genomic loci: *aflM/aflN*, *aflW/aflX*, *amdS*, *trpC*.

^c^ Isolate part of a subset for LD analysis in Figure 3.

^d^ AF- isolate groups with Geiser’s IB clade (25).

^e^ Isolate produces G_1_ < 0.5 μg/mL.

^f^ Isolate produces OMST < 40 μg/mL.

^g^ Isolate produces more B_2_ than B_1_.
