# Supplementary material for: Sexuality Generates Diversity in the Aflatoxin Gene Cluster: Evidence on a Global Scale
Source: PLoS Pathog. 2013 Aug 29;9(8):e1003574. doi: 10.1371/journal.ppat.1003574 (PMC3757046; doi:10.1371/journal.ppat.1003574)
Supplement: Table S6 — Aspergillus parasiticus isolates from Córdoba, Argentina. (DOC) [file ppat.1003574.s009.doc]

Table S6. *Aspergillus parasiticus* isolates from Córdoba, Argentina.

| **IC Strain** | ***MAT*** | **G_1_ (μg/mL)^a^** | **B_1_ (μg/mL)^a^** | **G_1_/B_1_** | **MLST^b^** |
| --- | --- | --- | --- | --- | --- |
| 480^c^ | 1 | 23.2 (5) | 379 (56) | 0.061 | H1 |
| 481 | 1 | 244.4 (33) | 128.5 (20) | 1.902 | - |
| 482 | 1 | 251.4 (26) | 128.2 (8) | 1.961 | - |
| 483 | 1 | 214.8 (32) | 121.5 (12) | 1.768 | - |
| 484 | 1 | 376.1 (9) | 98 (0.5) | 3.838 | H12 |
| 485^c^ | 1 | 16.1 (8) | 218.1 (79) | 0.074 | H1 |
| 486 | 1 | 293.5 (4) | 54.5 (0.2) | 5.385 | - |
| 487^c^ | 1 | 337.2 (22) | 99 (9) | 3.406 | H12 |
| 488 | 1 | 161.2 (48) | 35.2 (11) | 4.580 | - |
| 489 | 1 | 340.4 (32) | 104.2 (7) | 3.267 | H12 |
| 490 | 1 | 45.5 (11) | 23.4 (5) | 1.944 | H2 |
| 491^c^ | 1 | 374.7 (29) | 94.7 (3) | 3.957 | H12 |
| 492 | 1 | 33.4 (4) | 373.8 (58) | 0.089 | - |
| 493 | 1 | 314.2 (27) | 87.5 (9) | 3.591 | - |
| 494 | 2 | 215.6 (21) | 65.8 (13) | 3.277 | H5 |
| 495 | 1 | 20.4 (5) | 273.5 (52) | 0.075 | H1 |
| 496^c^ | 1 | 225 (12) | 111 (8) | 2.027 | H12 |
| 497 | 1 | 350.1 (50) | 73 (11) | 4.796 | H12 |
| 498 | 1 | 315 (41) | 64.3 (14) | 4.899 | - |
| 499 | 1 | 28.6 (6) | 255.1 (51) | 0.112 | H1 |
| 500^c^ | 1 | 26.6 (4) | 298.9 (49) | 0.089 | H1 |
| 501 | 1 | 344.3 (17) | 78.3 (4) | 4.397 | - |
| 502 | 1 | 341.1 (25) | 77.3 (3) | 4.413 | H12 |
| 503 | 1 | 333.8 (36) | 69.5 (9) | 4.803 | - |
| 504^c^ | 1 | 221.5 (12) | 115.4 (6) | 1.919 | H12 |
| 505 | 1 | 197.2 (32) | 122.7 (23) | 1.607 | H12 |
| 506 | 1 | 276 (11) | 77.2 (0.9) | 3.575 | H8 |
| 507 | 1 | 260 (21) | 155.3 (20) | 1.674 | H7 |
| 508^c^ | 1 | 310.4 (26) | 80.5 (9) | 3.856 | H12 |
| 509 | 1 | 288.1 (23) | 81.3 (4) | 3.544 | H12 |
| 510 | 1 | 345.9 (15) | 98.9 (3) | 3.498 | H12 |
| 511 | 1 | 22 (4) | 305.8 (41) | 0.072 | H1 |
| 512^c^ | 1 | 368.7 (19) | 181.7 (13) | 2.029 | H12 |
| 513 | 1 | 371.4 (26) | 187.3 (12) | 1.983 | H16 |
| 514 | 1 | 360.4 (33) | 202.1 (16) | 1.783 | H12 |
| 515 | 1 | 341.9 (42) | 166.6 (25) | 2.052 | - |
| 516^c^ | 1 | 354.8 (51) | 94.8 (12) | 3.743 | H12 |
| 517 | 1 | 160 (43) | 37.8 (8) | 4.233 | H3 |
| 518 | 1 | 139.6 (14) | 36.9 (6) | 3.783 | H3 |
| 519 | 1 | 19.1 (4) | 272.5 (29) | 0.070 | H11 |
| 520^c^ | 1 | 342.1 (24) | 88.9 (12) | 3.848 | H12 |
| 521 | 1 | 349.2 (36) | 89.8 (12) | 3.889 | H12 |
| 522 | 1 | 284.4 (28) | 77.6 (7) | 3.665 | H12 |
| 523 | 1 | 19.2 (2) | 264.9 (17) | 0.073 | H1 |
| 524^c^ | 1 | 24.8 (6) | 356.8 (45) | 0.069 | H17 |
| 525 | 1 | 25.1 (4) | 321.7 (52) | 0.078 | H1 |
| 526 | 2 | 1.1 (0.1) | 0.2 (0) | 5.500 | H6 |
| 527 | 1 | 205.2 (15) | 120.2 (11) | 1.707 | - |
| 528^c^ | 1 | 26.1 (5) | 348.6 (69) | 0.075 | H1 |
| 529 | 1 | 276.6 (1) | 78.2 (3) | 3.537 | H12 |
| 530 | 1 | 251.8 (29) | 73.8 (5) | 3.412 | H13 |
| 531 | 1 | 20.7 (2) | 313.7 (33) | 0.066 | H1 |
| 532^c^ | 1 | 290.2 (39) | 66 (9) | 4.397 | H12 |
| 533 | 1 | 292.5 (12) | 85.6 (5) | 3.417 | H12 |
| 534 | 1 | 30.6 (9) | 20.3 (8) | 1.507 | H14 |
| 535 | 1 | 20.5 (3) | 313.7 (17) | 0.065 | H15 |
| 536^c^ | 1 | 18.1 (0.5) | 297.5 (13) | 0.061 | H9 |
| 537 | 1 | 8.9 (0.3) | 193.7 (4) | 0.046 | H1 |
| 538 | 1 | 20.5 (3) | 291.8 (45) | 0.070 | H1 |
| 539 | 1 | 20.9 (2) | 309.2 (24) | 0.068 | H1 |
| 540^c^ | 1 | 306.3 (29) | 89.4 (16) | 3.426 | H12 |
| 541 | 1 | 311 (36) | 86.3 (6) | 3.604 | H12 |
| 542 | 1 | 330.2 (30) | 89.7 (5) | 3.681 | H12 |
| 543 | 1 | 325.9 (8) | 95.1 (5) | 3.427 | H12 |
| 544^c^ | 1 | 229.6 (37) | 133.9 (19) | 1.715 | H15 |
| 545 | 1 | 339.3 (21) | 96.8 (3) | 3.505 | H12 |
| 546 | 1 | 22.5 (3) | 315.1 (38) | 0.071 | H1 |
| 547 | 1 | 18.1 (3) | 232.3 (46) | 0.078 | H1 |
| 548^c^ | 1 | 34.6 (5) | 386.7 (50) | 0.089 | H15 |
| 549 | 1 | 385.1 (23) | 100.4 (7) | 3.836 | H12 |
| 550 | 1 | 24 (5) | 297.4 (44) | 0.081 | - |
| 551 | 1 | 26.3 (5) | 376.3 (43) | 0.070 | H10 |
| 552^c^ | 1 | 29.1 (2) | 337.7 (26) | 0.086 | H1 |
| 553 | 1 | 266.2 (5) | 129 (5) | 2.064 | H4 |
| 554 | 1 | 233 (11) | 112.7 (6) | 2.067 | H12 |
| 555 | 1 | 336.9 (16) | 88.2 (4) | 3.820 | H12 |
| 556^c^ | 1 | 29.3 (2) | 390.7 (24) | 0.075 | H16 |
| 557 | 1 | 280.3 (26) | 74.2 (3) | 3.778 | - |
| 558 | 1 | 20.4 (4) | 274.8 (52) | 0.074 | - |
| 559 | 1 | 29.1 (5) | 415.4 (58) | 0.070 | - |

^a^ AF concentration is based on average of three replicate cultures per isolate.

Number in parentheses is standard deviation.

^b^ Haplotypes based on four genomic loci: *aflM/aflN*, *aflW/aflX*, *amdS*, *trpC*.

^c^ Isolate part of a subset for LD analysis in Figure 3.
