# Supplementary material for: Sexuality Generates Diversity in the Aflatoxin Gene Cluster: Evidence on a Global Scale
Source: PLoS Pathog. 2013 Aug 29;9(8):e1003574. doi: 10.1371/journal.ppat.1003574 (PMC3757046; doi:10.1371/journal.ppat.1003574)
Supplement: Table S7 — Aspergillus parasiticus isolates from Georgia, United States. (DOC) [file ppat.1003574.s010.doc]

Table S7. *Aspergillus parasiticus* isolates from Georgia, United States.

| **IC Strain** | ***MAT*** | **G_1_ (μg/mL)^a^** | **B_1_ (μg/mL)^a^** | **G_1_/B_1_** | **MLST^b^** |
| --- | --- | --- | --- | --- | --- |
| 1 | 1 | 94.5 (6) | 111.1 (7) | 0.851 | H1 |
| 2^c^ | 1 | 72.5 (6) | 94.9 (9) | 0.764 | H1 |
| 3 | 1 | 71.2 (15) | 91.7 (9) | 0.776 | - |
| 4 | 1 | 91.9 (16) | 105.8 (19) | 0.869 | - |
| 5 | 1 | 60.4 (3) | 94.1 (0.7) | 0.642 | H1 |
| 6 | 1 | 54.4 (4) | 75.7 (8) | 0.719 | - |
| 7 | 1 | 53.4 (4) | 75.3 (3) | 0.709 | H1 |
| 8 | 1 | 75.7 (9) | 91.6 (11) | 0.826 | H1 |
| 9 | 1 | 59.3 (10) | 78.8 (7) | 0.753 | - |
| 10 | 1 | 72.2 (13) | 88 (11) | 0.821 | H1 |
| 11^c^ | 1 | 79.8 (8) | 91.7 (3) | 0.870 | H1 |
| 12 | 1 | 81.9 (36) | 104 (29) | 0.788 | H1 |
| 13 | 1 | 68 (18) | 83.9 (11) | 0.811 | H1 |
| 14 | 1 | 79.1 (12) | 94.3 (13) | 0.839 | H1 |
| 15^c^ | 1 | 74.5 (12) | 91.1 (15) | 0.818 | H1 |
| 16 | 1 | 57.1 (18) | 79.5 (7) | 0.718 | - |
| 17 | 1 | 98.1 (46) | 114.9 (51) | 0.854 | H1 |
| 18 | 1 | 168.1 (21) | 119.9 (10) | 1.402 | H16 |
| 19 | 1 | 102.6 (35) | 69.3 (20) | 1.481 | H6 |
| 20 | 1 | 172.8 (9) | 105.9 (4) | 1.632 | - |
| 21 | 1 | 195.9 (36) | 130.2 (25) | 1.505 | H16 |
| 22 | 1 | 149.4 (16) | 105.7 (2) | 1.413 | H16 |
| 23 | 1 | 169.9 (15) | 103.1 (5) | 1.648 | H16 |
| 24 | 1 | 161.1 (13) | 123 (6) | 1.310 | H16 |
| 25^c^ | 1 | 158 (37) | 112.3 (24) | 1.407 | H16 |
| 26 | 1 | 202.5 (35) | 133.3 (14) | 1.519 | H16 |
| 27 | 1 | 220.7 (31) | 141.2 (33) | 1.563 | H16 |
| 28 | 1 | 218.9 (18) | 155.7 (1) | 1.406 | - |
| 29 | 1 | 200.4 (7) | 140.9 (14) | 1.422 | H16 |
| 30 | 1 | 170.2 (63) | 130.8 (38) | 1.301 | - |
| 32 | 1 | 217.8 (23) | 132 (13) | 1.650 | H1 |
| 33 | 2 | 117.4 (26) | 86.6 (12) | 1.356 | H1 |
| 34 | 1 | 227.9 (5) | 136.1 (9) | 1.675 | H1 |
| 35 | 1 | 212.6 (7) | 114.5 (2) | 1.857 | H12 |
| 36 | 1 | 223.6 (44) | 135.2 (25) | 1.654 | H1 |
| 37 | 1 | 288.1 (127) | 176.1 (85) | 1.636 | H1 |
| 38^c^ | 2 | 95.8 (7) | 91.4 (8) | 1.048 | H1 |
| 39 | 1 | 231 (39) | 137.4 (14) | 1.681 | H1 |
| 40 | 1 | 195.5 (2) | 141.4 (15) | 1.383 | H1 |
| 41 | 1 | 230.8 (31) | 143.6 (10) | 1.607 | - |
| 42 | 1 | 262.6 (21) | 127.2 (3) | 2.065 | H1 |
| 43^c^ | 1 | 36.2 (11) | 376.9 (70) | 0.096 | - |
| 44 | 1 | 15.9 (1) | 120.3 (14) | 0.132 | H15 |
| 45 | 1 | 26.5 (6) | 270.8 (30) | 0.098 | - |
| 46 | 1 | 28.9 (6) | 310.2 (66) | 0.093 | H15 |
| 47 | 1 | 30.7 (3) | 319.7 (10) | 0.096 | H5 |
| 48 | 1 | 25.2 (2) | 271.3 (17) | 0.093 | H15 |
| 49 | 1 | 28.4 (4) | 273.7 (20) | 0.104 | H15 |
| 50 | 1 | 21.8 (4) | 217.5 (22) | 0.100 | H15 |
| 51 | 1 | 20.3 (3) | 203.9 (20) | 0.100 | H15 |
| 52 | 1 | 158.8 (52) | 144.5 (52) | 1.099 | H1 |
| 53 | 2 | 196.1 (16) | 161.2 (10) | 1.217 | H1 |
| 54 | 1 | 197.4 (11) | 168.1 (12) | 1.174 | H4 |
| 55 | 1 | 198.7 (28) | 166.8 (31) | 1.191 | H1 |
| 56^c^ | 1 | 203.1 (13) | 176.6 (12) | 1.150 | H1 |
| 57 | 2 | 243.5 (13) | 145.2 (15) | 1.677 | - |
| 58 | 2 | 326.2 (17) | 158.9 (21) | 2.053 | H13 |
| 59^c^ | 2 | 229.3 (23) | 124.1 (11) | 1.848 | H13 |
| 60 | 2 | 231.3 (8) | 135.5 (7) | 1.707 | H13 |
| 61^c^ | 1 | 190.9 (41) | 142.2 (34) | 1.343 | H14 |
| 62 | 1 | 178.2 (9) | 132.5 (10) | 1.345 | H14 |
| 63 | 1 | 195 (23) | 140.2 (21) | 1.391 | H14 |
| 64 | 1 | 169.7 (25) | 130 (26) | 1.305 | H3 |
| 65^c,e^ | 2 | 0.0 (0) | 0.0 (0) | 0.0 | H9 |
| 66 | 2 | 0.0 (0) | 0.0 (0) | 0.0 | H9 |
| 67^c,e^ | 2 | 143.2 (8) | 97.5 (7) | 1.469 | H8 |
| 68 | 1 | 161.2 (17) | 137 (17) | 1.177 | H7 |
| 69^c^ | 2 | 85.1 (62) | 59.3 (44) | 1.435 | H1 |
| 70^c^ | 1 | 474.6 (43) | 236.6 (32) | 2.006 | H11 |
| 71^c,d^ | 2 | 0.0 (0) | 0.0 (0) | 0.0 | H9 |
| 72^c^ | 2 | 253.4 (34) | 167.3 (15) | 1.515 | H13 |
| 73^c^ | 1 | 377.6 (22) | 73.7 (4) | 5.124 | H2 |
| 74^c^ | 1 | 189.2 (9) | 97.6 (5) | 1.939 | H13 |
| 75^c^ | 1 | 203 (6) | 142 (2) | 1.430 | H1 |
| 76^c,d^ | 2 | 0.0 (0) | 0.0 (0) | 0.0 | H10 |

^a^ AF concentration is based on average of three replicate cultures per isolate.

Number in parentheses is standard deviation.

^b^ Haplotypes based on four genomic loci: *aflM/aflN*, *aflW/aflX*, *amdS*, *trpC*.

^c^ Isolate part of a subset for LD analysis in Figure 3.

^d^ Isolate produces OMST < 200 μg/mL.

^e^ Isolate produces OMST > 200 μg/mL.
