# Supplementary material for: Sexuality Generates Diversity in the Aflatoxin Gene Cluster: Evidence on a Global Scale
Source: PLoS Pathog. 2013 Aug 29;9(8):e1003574. doi: 10.1371/journal.ppat.1003574 (PMC3757046; doi:10.1371/journal.ppat.1003574)
Supplement: Table S8 — Aspergillus parasiticus isolates from Queensland, Australia. (DOC) [file ppat.1003574.s011.doc]

Table S8. *Aspergillus parasiticus* isolates from Queensland, Australia.

| **IC Strain** | ***MAT*** | **G_1_ (μg/mL)^a^** | **B_1_ (μg/mL)^a^** | **G_1_/B_1_** | **MLST^b^** |
| --- | --- | --- | --- | --- | --- |
| 800^c^ | 2 | 149 (42) | 51.1 (15) | 2.916 | H1 |
| 801 | 2 | 141.1 (46) | 46.3 (13) | 3.048 | H1 |
| 802 | 2 | 117.8 (62) | 42.8 (24) | 2.752 | - |
| 803 | 2 | 162.3 (26) | 56.5 (6) | 2.873 | - |
| 804 | 2 | 110.5 (64) | 39.4 (23) | 2.805 | H1 |
| 805 | 2 | 111.7 (18) | 41.5 (7) | 2.692 | H1 |
| 806 | 2 | 267.1 (80) | 50 (14) | 5.342 | H7 |
| 807 | 2 | 134.6 (22) | 175.5 (37) | 0.767 | H20 |
| 808^c^ | 2 | 162 (32) | 181.3 (30) | 0.894 | H14 |
| 809^d^ | 2 | 0.0 (0) | 0.0 (0) | 0.0 | H19 |
| 810 | 2 | 74 (3) | 107.5 (10) | 0.688 | - |
| 811^c^ | 1 | 64.2 (6) | 20.5 (2) | 3.132 | H10 |
| 812 | 1 | 68 (17) | 20.7 (3) | 3.285 | - |
| 813 | 1 | 51.3 (22) | 18.5 (5) | 2.773 | H8 |
| 814 | 2 | 86.3 (10) | 129.8 (9) | 0.665 | H21 |
| 815 | 2 | 53 (6) | 17.9 (2) | 2.961 | - |
| 816^c^ | 2 | 165.6 (58) | 84.1 (40) | 1.969 | H26 |
| 817 | 1 | 211 (23) | 103.7 (13) | 2.035 | - |
| 818 | 2 | 187.6 (4) | 86.6 (6) | 2.166 | - |
| 819 | 1 | 220.3 (63) | 102.1 (39) | 2.158 | - |
| 820 | 2 | 174.8 (13) | 80.4 (7) | 2.174 | - |
| 821 | 2 | 173.5 (16) | 87.1 (5) | 1.992 | - |
| 822^c^ | 2 | 169.1 (30) | 74.5 (19) | 2.270 | H25 |
| 823 | 2 | 121.3 (12) | 165.6 (9) | 0.732 | - |
| 824^c^ | 1 | 185.5 (28) | 112.1 (12) | 1.655 | H23 |
| 825 | 1 | 142.9 (49) | 362.9 (131) | 0.394 | H18 |
| 826 | 1 | 166.4 (9) | 117.7 (6) | 1.414 | - |
| 827 | 2 | 0.011 (0) | 0.012 (0) | 0.917 | - |
| 828^c^ | 2 | 196.1 (39) | 109.8 (23) | 1.786 | H17 |
| 829 | 1 | 357.7 (47) | 279.3 (40) | 1.281 | - |
| 830 | 2 | 174.5 (41) | 98 (19) | 1.781 | - |
| 831 | 1 | 112.5 (24) | 29.2 (5) | 3.853 | - |
| 832^c^ | 2 | 7.8 (1) | 372.3 (37) | 0.021 | H11 |
| 833 | 2 | 16.1 (3) | 58.6 (8) | 0.275 | - |
| 834 | 1 | 43.2 (12) | 14.9 (4) | 2.899 | - |
| 835 | 1 | 225.1 (9) | 195.4 (22) | 1.152 | H25 |
| 836^c^ | 1 | 59.4 (15) | 21.3 (3) | 2.789 | H12 |
| 837 | 2 | 124 (14) | 43.6 (4) | 2.844 | H2 |
| 838 | 1 | 165.5 (41) | 76.4 (21) | 2.166 | - |
| 839 | 2 | 218.4 (43) | 103.3 (23) | 2.114 | H3 |
| 840^c^ | 2 | 183 (19) | 58.8 (5) | 3.112 | H1 |
| 841 | 2 | 182.3 (46) | 94.8 (27) | 1.923 | - |
| 842 | 1 | 159.1 (28) | 49 (8) | 3.247 | - |
| 843 | 1 | 209 (10) | 160.2 (8) | 1.305 | - |
| 844^c^ | 1 | 216.2 (31) | 148.9 (22) | 1.452 | H22 |
| 845 | 1 | 58.6 (18) | 18.7 (3) | 3.134 | - |
| 846 | 2 | 107.1 (28) | 37.1 (10) | 2.887 | - |
| 847 | 1 | 151.1 (12) | 73 (17) | 2.070 | - |
| 848^c^ | 2 | 338.6 (147) | 122.2 (50) | 2.771 | H11 |
| 849 | 1 | 55.5 (9) | 52.5 (6) | 1.057 | - |
| 850 | 1 | 39.7 (13) | 41.5 (12) | 0.957 | - |
| 851 | 1 | 209.1 (10) | 129.5 (9) | 1.615 | H16 |
| 852 | 2 | 166.2 (9) | 52.5 (2) | 3.166 | - |
| 853^c^ | 2 | 84.7 (9) | 127.6 (9) | 0.664 | H21 |
| 854^c^ | 2 | 163.4 (18) | 67.6 (5) | 2.417 | H27 |
| 855 | 2 | 141.8 (16) | 56.9 (9) | 2.492 | - |
| 856 | 2 | 159.3 (8) | 59.4 (0.9) | 2.682 | - |
| 857 | 2 | 152 (25) | 54.2 (13) | 2.804 | - |
| 858 | 1 | 125.4 (18) | 88.4 (14) | 1.419 | - |
| 859 | 2 | 151.8 (13) | 57.4 (6) | 2.645 | - |
| 860^c^ | 2 | 43.8 (15) | 15 (4) | 2.920 | H9 |
| 861 | 2 | 51.3 (1) | 16 (1) | 3.206 | - |
| 862 | 1 | 164.1 (32) | 421.3 (61) | 0.390 | - |
| 863 | 2 | 123.9 (11) | 47.3 (2) | 2.619 | H6 |
| 864^c^ | 1 | 201 (11) | 208.8 (24) | 0.963 | H14 |
| 865 | 1 | 69.1 (45) | 81.9 (37) | 0.844 | - |
| 866 | 2 | 84.7 (11) | 105.8 (13) | 0.801 | - |
| 867 | 2 | 91 (8) | 112.8 (9) | 0.807 | H4 |
| 868^c^ | 1 | 179.5 (17) | 132.8 (8) | 1.352 | H24 |
| 869 | 1 | 193.2 (18) | 116.8 (22) | 1.654 | - |
| 870 | 1 | 26.3 (8) | 12.7 (3) | 2.071 | - |
| 871 | - | 185.1 (42) | 107 (29) | 1.730 | - |
| 872^c^ | 1 | 201.8 (18) | 237.2 (15) | 0.851 | H15 |
| 873 | 2 | 165.4 (9) | 115.8 (3) | 1.428 | - |
| 874 | 2 | 423.6 (33) | 153.9 (11) | 2.752 | - |
| 875 | 1 | 195.3 (9) | 216.7 (13) | 0.901 | H5 |
| 876^c^ | 2 | 52.9 (7) | 17 (3) | 3.112 | H13 |
| 877 | 2 | 68.5 (7) | 20.3 (2) | 3.374 | - |
| 878 | 2 | 383.8 (26) | 141.8 (13) | 2.707 | - |
| 879 | 2 | 437.3 (24) | 154.1 (10) | 2.838 | - |

^a^ AF concentration is based on average of three replicate cultures per isolate.

Number in parentheses is standard deviation.

^b^ Haplotypes based on four genomic loci: *aflM/aflN*, *aflW/aflX*, *amdS*, *trpC*.

^c^ Isolate part of a subset for LD analysis in Figure 3.

^d^ Isolate produces OMST < 200 μg/mL.
