# Supplementary material for: Sexuality Generates Diversity in the Aflatoxin Gene Cluster: Evidence on a Global Scale
Source: PLoS Pathog. 2013 Aug 29;9(8):e1003574. doi: 10.1371/journal.ppat.1003574 (PMC3757046; doi:10.1371/journal.ppat.1003574)
Supplement: Table S9 — Aspergillus flavus S and A. minisclerotigenes isolates from Queensland, Australia. (DOC) [file ppat.1003574.s012.doc]

Table S9. *Aspergillus flavus* S and *A. minisclerotigenes* isolates from Queensland, Australia.

| **IC Strain** | ***MAT*** | **G_1_ (μg/mL)^a^** | **B_1_ (μg/mL)^a^** | **G_1_/B_1_** | **MLST^b^** |
| --- | --- | --- | --- | --- | --- |
| *A. flavus* S | | | | | |
| 723^c,d^ | 2 | 0.0 (0) | 76.2 (0.7) | 0.0 | H17 |
| 724 | - | 0.0 (0) | 0.0 (0) | 0.0 | - |
| 725^d^ | 2 | 0.0 (0) | 108 (46) | 0.0 | H17 |
| 727^d^ | 2 | 0.0 (0) | 59.4 (13) | 0.0 | H17 |
| 728^d^ | 2 | 0.0 (0) | 82.3 (14) | 0.0 | H17 |
| 729^d^ | 2 | 0.0 (0) | 61.5 (2) | 0.0 | H17 |
| 732^d^ | 1 | 0.0 (0) | 29.6 (1.2) | 0.0 | H17 |
| 736^d^ | 2 | 0.0 (0) | 82.9 (17) | 0.0 | H17 |
| 737^d^ | 2 | 0.0 (0) | 84.3 (20) | 0.0 | H17 |
| 738 | 2 | 0.0 (0) | 62 (9) | 0.0 | - |
| 739 | 2 | 0.0 (0) | 65 (4) | 0.0 | - |
| 740 | - | 0.0 (0) | 93.7 (16.4) | 0.0 | - |
| 741^d^ | 2 | 0.0 (0) | 67.9 (16) | 0.0 | H21 |
| 743^d^ | 2 | 0.0 (0) | 23.8 (4) | 0.0 | H2 |
| 746 | - | 0.0 (0) | 27.6 (8.9) | 0.0 | - |
| 748^d^ | 2 | 0.0 (0) | 78.9 (9) | 0.0 | H17 |
| 749^d^ | 2 | 0.0 (0) | 73.7 (22) | 0.0 | H17 |
| 750 | - | 0.0 (0) | 92.2 (9.9) | 0.0 | - |
| 751^d^ | 2 | 0.0 (0) | 14.9 (2) | 0.0 | H18 |
| 752 | - | 0.0 (0) | 0.0 (0) | 0.0 | - |
| 753 | 2 | 0.0 (0) | 30.4 (4) | 0.0 | H11 |
| 754 | - | 0.0 (0) | 34.7 (6.2) | 0.0 | - |
| 755^c,d^ | 2 | 0.0 (0) | 9.9 (2) | 0.0 | H19 |
| 756 | - | 0.0 (0) | 6.9 (2.2) | 0.0 | - |
| 758^d^ | 2 | 0.0 (0) | 6.9 (0.7) | 0.0 | H13 |
| 760^c^ | 2 | 0.0 (0) | 78.3 (18) | 0.0 | H11 |
| 761 | - | 0.0 (0) | 38.4 (5.1) | 0.0 | - |
| 762^d^ | 2 | 0.0 (0) | 49.5 (8) | 0.0 | H17 |
| 764 | - | 0.0 (0) | 18 (3.3) | 0.0 | - |
| 765 | - | 0.0 (0) | 36 (8.3) | 0.0 | - |
| 767 | - | 0.0 (0) | 29.3 (11.2) | 0.0 | - |
| 768^c,d^ | 2 | 0.0 (0) | 13.9 (7) | 0.0 | H19 |
| 770^d^ | 2 | 0.0 (0) | 57.6 (7) | 0.0 | H14 |
| 772 | - | 0.0 (0) | 39.2 (5.3) | 0.0 | - |
| 777^d^ | 2 | 0.0 (0) | 12.8 (3) | 0.0 | H16 |
| 779^c,d^ | 2 | 0.0 (0) | 113 (12) | 0.0 | H17 |
| 780^d^ | 2 | 0.0 (0) | 76.6 (20) | 0.0 | H15 |
| 781 | 2 | 0.0 (0) | 13.4 (3) | 0.0 | - |
| 783 | - | 0.0 (0) | 34.2 (14.6) | 0.0 | - |
| 784 | 2 | 0.0 (0) | 13.7 (3) | 0.0 | - |
| 785^d^ | 2 | 0.0 (0) | 34.7 (23) | 0.0 | H3 |
| 786^d^ | 2 | 0.0 (0) | 31.8 (16) | 0.0 | H9 |
| 787^d^ | 2 | 0.0 (0) | 27.6 (5) | 0.0 | H4 |
| 789 | - | 0.0 (0) | 18.1 (4.9) | 0.0 | - |
| 790^c,d^ | 1 | 0.0 (0) | 16.5 (2) | 0.0 | H20 |
| 791^d^ | 1 | 0.0 (0) | 13.8 (5) | 0.0 | H6 |
| 792^d^ | 2 | 0.0 (0) | 73 (9) | 0.0 | H8 |
| 793 | 1 | 0.0 (0) | 0.0 (0) | 0.0 | H12 |
| 795 | - | 0.0 (0) | 11.6 (2.8) | 0.0 | - |
| 796^d^ | 1 | 0.0 (0) | 130 (16) | 0.0 | H17 |
| 797^c,d^ | 2 | 0.0 (0) | 65.6 (8) | 0.0 | H17 |
| 798^d^ | 2 | 0.0 (0) | 14.2 (3) | 0.0 | H5 |
| 799^d^ | 1 | 0.0 (0) | 5.1 (2) | 0.0 | H10 |
| *A. minisclerotigenes* | | | | | |
| 720^c,e^ | 1 | 11.1 (2) | 4.2 (0.4) | 2.643 | H1 |
| 721 | - | 12.3 (1.1) | 4.7 (0.6) | 2.617 | - |
| 726 | - | 7.9 (2.6) | 4 (1.3) | 1.975 | - |
| 722^e^ | 2 | 1.3 (0.4) | 0.8 (0.2) | 1.625 | - |
| 730 | - | 4.5 (1.8) | 2.1 (0.7) | 2.143 | - |
| 731^e^ | 1 | 1.3 (0.1) | 1.0 (0.2) | 1.3 | H22 |
| 733^d,e^ | 1 | 2.0 (0.2) | 1.3 (0.1) | 1.538 | H23 |
| 734^e^ | 1 | 2.1 (0.5) | 1.2 (0.3) | 1.75 | H10 |
| 735^d,e^ | 1 | 1.6 (0.1) | 1.5 (0.2) | 1.6 | H23 |
| 742^e^ | 1 | 5.9 (1) | 3.2 (0.5) | 1.844 | H25 |
| 744^c,e^ | 1 | 4.7 (1) | 3.1 (0.5) | 1.516 | H24 |
| 747 | - | 3.5 (0.3) | 2.7 (0.2) | 1.296 | - |
| 757 | - | 2.7 (0.4) | 2.2 (0.5) | 1.227 | - |
| 759 | - | 0.9 (0.1) | 0.8 (0.1) | 1.125 | - |
| 763 | - | 0.2 (0.1) | 0.2 (0.1) | 1.0 | - |
| 766 | - | 3.6 (1.8) | 1.8 (0.4) | 2.0 | - |
| 769 | - | 18.4 (14.9) | 5.2 (2.4) | 3.538 | - |
| 771 | - | 5 (1.8) | 2.7 (0.5) | 1.852 | - |
| 773 | - | 5.8 (0.9) | 2.9 (0.5) | 2.0 | - |
| 774^e^ | 2 | 8.4 (7) | 3.5 (2) | 2.4 | - |
| 775^e^ | 2 | 5.9 (3) | 3.0 (1) | 1.967 | - |
| 776^e^ | 2 | 2.8 (0.1) | 1.6 (0.3) | 1.75 | - |
| 778 | 1 | 2.6 (0.3) | 1.4 (0.1) | 1.857 | - |
| 782 | 1 | 7.8 (1.8) | 4 (0.6) | 1.95 | - |
| 788^d,e^ | 2 | 0.3 (0) | 0.4 (0) | 0.75 | H7 |
| 794 | - | 3.4 (1) | 1.8 (0.4) | 1.889 | - |

^a^ AF concentration is based on average of three replicate cultures per isolate.

Number in parentheses is standard deviation.

^b^ Haplotypes based on four genomic loci: *aflM/aflN*, *aflW/aflX*, *amdS*, *trpC*.

^c^ Isolate part of a subset for LD analysis in Figure 3.

^d^ Isolate shares haplotype with Geiser’s group II strains (25) based on *amdS* and *trpC*.

^e^ Isolate produces OMST < 0.5 μg/mL.
