# Supplementary material for: Sexuality Generates Diversity in the Aflatoxin Gene Cluster: Evidence on a Global Scale
Source: PLoS Pathog. 2013 Aug 29;9(8):e1003574. doi: 10.1371/journal.ppat.1003574 (PMC3757046; doi:10.1371/journal.ppat.1003574)
Supplement: Table S10 — Aspergillus flavus S and A. minisclerotigenes isolates from Littoral, Benin. (DOC) [file ppat.1003574.s013.doc]

Table S10. *Aspergillus flavus* S and *A. minisclerotigenes* isolates from Littoral, Benin.

| **IC Strain** | ***MAT*** | **G_1_ (μg/mL)^a^** | **B_1_ (μg/mL)^a^** | **G_1_/B_1_** | **MLST^b^** |
| --- | --- | --- | --- | --- | --- |
| *A. flavus* S | | | | | |
| 1152^c^ | 2 | 0.0 (0) | 16.6 (1) | 0.0 | H2 |
| 1153^c^ | 1 | 0.0 (0) | 46.1 (19) | 0.0 | H15 |
| *A. minisclerotigenes* | | | | | |
| 1110 | - | 52.7 (10.1) | 50.1 (15) | 1.053 | - |
| 1111 | - | 9.0 (2.8) | 10.1 (3.3) | 0.888 | - |
| 1112^c^ | 1 | 129.6 (9) | 33.5 (5) | 3.874 | H1 |
| 1113^c^ | 1 | 13.4 (0.7) | 10.7 (0.4) | 1.258 | H3 |
| 1114 | 2 | 96.7 (3) | 42.5 (3) | 2.272 | - |
| 1115 | 2 | 20.3 (2) | 6.59 (0.6) | 3.082 | - |
| 1116 | - | 17.8 (3.5) | 5.6 (1) | 3.175 | - |
| 1117 | 2 | 25.7 (7) | 12.8 (3) | 2.008 | H9 |
| 1118 | 1 | 17.7 (3) | 13.9 (2) | 1.269 | H11 |
| 1119 | 1 | 18.4 (2) | 13 (0.6) | 1.411 | H13 |
| 1120^c^ | 1 | 18.9 (0.9) | 15 (0.6) | 1.26 | H3 |
| 1121 | 1 | 12.5 (1) | 9.3 (0.7) | 1.351 | H3 |
| 1122 | 2 | 26 (3) | 20.6 (2) | 1.258 | - |
| 1123 | 2 | 33.8 (3) | 17 (0.9) | 1.996 | - |
| 1124 | 2 | 11.3 (3) | 15.7 (3) | 0.722 | - |
| 1125 | - | 32.8 (9.4) | 17.5 (1.7) | 1.882 | - |
| 1126 | - | 9.5 (1.1) | 16.5 (2.2) | 0.576 | - |
| 1127 | - | 17 (6.3) | 21.1 (5.5) | 0.805 | - |
| 1128 | - | 22.5 (0.8) | 6.5 (0.4) | 3.474 | - |
| 1129 | 1 | 9.7 (0.8) | 14 (3) | 0.692 | - |
| 1130 | 1 | 22.9 (2) | 27 (2) | 0.847 | - |
| 1131 | 1 | 11.9 (3) | 18.8 (4) | 0.634 | - |
| 1132 | 1 | 8.2 (2) | 8.3 (1) | 0.997 | - |
| 1133 | 1 | 14.3 (2) | 11.5 (1) | 1.247 | H14 |
| 1134 | 2 | 15.5 (3) | 12.2 (2) | 1.27 | H10 |
| 1135 | 1 | 17.9 (2) | 14.1 (2) | 1.27 | H3 |
| 1136 | 2 | 20 (2) | 5.6 (0.7) | 3.574 | - |
| 1137 | - | 21 (2.2) | 6 (0.7) | 3.523 | - |
| 1138 | - | 20.7 (0.2) | 5.9 (0.1) | 3.503 | - |
| 1139 | - | 20.6 (1.3) | 5.7 (0.7) | 3.627 | - |
| 1140^c^ | 2 | 17.8 (1) | 4.7 (0.3) | 3.806 | H4 |
| 1141 | 1 | 16.5 (2) | 12.5 (1) | 1.314 | H12 |
| 1142^c^ | 2 | 116 (3) | 45.8 (2) | 2.534 | H8 |
| 1143 | 2 | 15.6 (0.3) | 4.7 (0.2) | 3.326 | - |
| 1144 | 2 | 30.3 (3) | 14.8 (2) | 2.051 | H5 |
| 1145 | 2 | 12.3 (2) | 15.6 (1) | 0.787 | H16 |
| 1146 | 2 | 33.4 (3) | 15.1 (2) | 2.209 | H7 |
| 1147 | 2 | 9 (0.5) | 13.9 (2) | 0.652 | H17 |
| 1148 | 2 | 36 (5) | 16.4 (2) | 2.205 | H7 |
| 1149 | 2 | 39.9 (8) | 18.5 (3) | 2.159 | H6 |
| 1150^c^ | 2 | 47.3 (7) | 21.1 (3) | 2.241 | H7 |
| 1151 | 2 | 10 (1) | 14.6 (2) | 0.685 | H17 |

^a^ AF concentration is based on average of three replicate cultures per isolate.

Number in parentheses is standard deviation.

^b^ Haplotypes based on four genomic loci: *aflM/aflN*, *aflW/aflX*, *amdS*, *trpC*.

^c^ Isolate part of a subset for LD analysis in Figure 3.
