# Supplementary material for: Sexuality Generates Diversity in the Aflatoxin Gene Cluster: Evidence on a Global Scale
Source: PLoS Pathog. 2013 Aug 29;9(8):e1003574. doi: 10.1371/journal.ppat.1003574 (PMC3757046; doi:10.1371/journal.ppat.1003574)
Supplement: Table S11 — Aspergillus flavus S and A. minisclerotigenes isolates from Córdoba, Argentina. (DOC) [file ppat.1003574.s014.doc]

Table S11. *Aspergillus flavus* S and *A. minisclerotigenes* isolates from Córdoba, Argentina.

| **IC Strain** | ***MAT*** | **G_1_ (μg/mL)^a^** | **B_1_ (μg/mL)^a^** | **G_1_/B_1_** | **MLST^b^** |
| --- | --- | --- | --- | --- | --- |
| ***A. flavus* S** | | | | | |
| 476 | 2 | 0.0 (0) | 157.9 (17) | 0.0 | H2 |
| 479 | 2 | 0.0 (0) | 80 (17) | 0.0 | H2 |
| ***A. minisclerotigenes*** | | | | | |
| 477 | 1 | 6.35 (1) | 2.35 (0.1) | 2.702 | H3 |
| 478^c^ | 1 | 7.06 (0.8) | 1.99 (0.3) | 3.548 | H1 |

^a^ AF concentration is based on average of three replicate cultures per isolate.

Number in parentheses is standard deviation.

^b^ Haplotypes based on four genomic loci: *aflM/aflN*, *aflW/aflX*, *amdS*, *trpC*.

^c^ Isolate shares haplotype with Geiser’s group II (25) strains based on *amdS* and *trpC*.
